# Sleep as a random walk: a super-statistical analysis of EEG data across sleep stages

**DOI:** 10.1038/s42003-021-02912-6

**Published:** 2021-12-10

**Authors:** Claus Metzner, Achim Schilling, Maximilian Traxdorf, Holger Schulze, Patrick Krauss

**Affiliations:** 1 Neuroscience Lab, Experimental Otolaryngology, University Hospital Erlangen, Erlangen, Germany; 2 Laboratory of Sensory and Cognitive Neuroscience, Aix-Marseille University, Marseille, France; 3 Cognitive Computational Neuroscience Group, Friedrich-Alexander University Erlangen-Nuremberg, Nuremberg, Germany; 4 Department of Otorhinolaryngology, Paracelsus Medical University, Nuremberg, Germany; 5 Pattern Recognition Lab, Friedrich-Alexander University Erlangen-Nuremberg, Nuremberg, Germany

**Keywords:** Sleep, Statistical methods

## Abstract

In clinical practice, human sleep is classified into stages, each associated with different levels of muscular activity and marked by characteristic patterns in the EEG signals. It is however unclear whether this subdivision into discrete stages with sharply defined boundaries is truly reflecting the dynamics of human sleep. To address this question, we consider one-channel EEG signals as heterogeneous random walks: stochastic processes controlled by hyper-parameters that are themselves time-dependent. We first demonstrate the heterogeneity of the random process by showing that each sleep stage has a characteristic distribution and temporal correlation function of the raw EEG signals. Next, we perform a super-statistical analysis by computing hyper-parameters, such as the standard deviation, kurtosis, and skewness of the raw signal distributions, within subsequent 30-second epochs. It turns out that also the hyper-parameters have characteristic, sleep-stage-dependent distributions, which can be exploited for a simple Bayesian sleep stage detection. Moreover, we find that the hyper-parameters are not piece-wise constant, as the traditional hypnograms would suggest, but show rising or falling trends within and across sleep stages, pointing to an underlying continuous rather than sub-divided process that controls human sleep. Based on the hyper-parameters, we finally perform a pairwise similarity analysis between the different sleep stages, using a quantitative measure for the separability of data clusters in multi-dimensional spaces.

## Abstract (web summary)

To improve our understand of how EEG activity reflects the dynamics of human sleep, Metzner et al. use human EEG data and superstatistical analysis to demonstrate that each sleep stage has a characteristic distribution and temporal correlation function of raw EEG signals. They also show that the hyper-parameters controlling the EEG signals have characteristic, sleep-stage-dependent distributions, which can be exploited for a simple Bayesian sleep stage detection.

## Introduction

Many complex systems, such as the earth’s crust, the weather, biological organisms, or the stock market, show continuous fluctuations of their internal state variables, even in the absence of external perturbations. The underlying processes can often be quantified in the form of multivariate time series and a mathematical analysis of the time series can be used to predict future states of the system, or simply to better understand its internal dynamics^1–3^.

Although in simple physical systems, state variables fluctuate around a fixed mean value and with a fixed variance (as in the case of local pressure variations in a gas at equilibrium), complex systems often have multiple dynamical attractors^4^, i.e., a set of qualitatively different modes of behavior, between which the system will occasionally switch. Such transitions typically show up in the time series by a sudden (or gradual) change of the statistical properties of the fluctuating state variables.

A typical example of such mode-switching behavior is the sleep cycle in humans and other mammals, where the brain is passing through a sequence of seemingly distinct sleep stages^5^. In this case, multi-channel electroencephalographic (EEG) recordings offer a convenient way to quantify the ongoing changes in the brain over long periods, but also with high temporal resolution^6,7^. The momentary amplitudes of an N-channel EEG recording represent a point in an N-dimensional state space and the ongoing time series of vectorial amplitudes defines a random walk within this high-dimensional space.

It is then a natural hypothesis that each sleep stage corresponds to a different cluster within EEG state space, and that the trajectory of the random walk moves to the corresponding cluster whenever a new sleep stage is entered. Indeed, we have confirmed this hypothesis in former work^8^, where we applied a previously developed method for analyzing and comparing spatiotemporal cortical activation patterns^9^. In this context, we have also analyzed the micro-structure of cortical activity during sleep and found that it reflects respiratory events and the state of daytime vigilance^10^. Moreover, we have developed a general method to quantify the separability of point clusters in high-dimensional state spaces^11^.

In principle, the existence of sleep-stage-related clusters within the EEG state space could be exploited for an automatic detection of these stages, based only on the momentary multi-channel amplitudes, or on short-time averages of those. However, modern methods of automatic sleep-stage detection are usually based on sliding time windows of a larger width, so that the algorithm can also make use of temporal features in the EEG data that are characteristic for different sleep stages (such as sleep spindles or K-complexes)^12,13^. In this case, it is not even necessary to record a large number of EEG channels. Indeed, we have shown that reliable sleep-stage detection is even possible based on a single channel^14^, thanks to the remarkable ability of machine learning systems to extract those features from the data that are most relevant for the classification task.

In this work, we continue our investigation of single-channel EEG data during human sleep. However, our present focus is not on the further improvement of automatic sleep-stage detection, but on a more fundamental description of the statistical properties of EEG data, seen as a temporally heterogeneous random walk. In particular, we investigate how the random walks momentary statistical properties, also called hyper-parameters, are changing during and across sleep stages. Our approach is based on the method of super-statistical analysis^15–17^, which we have originally developed to analyze the random migration patterns of individual cancer cells^18^, revealing that their average migration speed, the directional persistence of the cell trajectories, and other hyper-parameters are time-dependent, reflecting internal mode changes such as the cell cycle. In subsequent work, we have demonstrated that the method can also be used to extract and model gradual or abrupt hyper-parameter changes in other complex dynamical systems, such as the climate or the stock market^19^.

In the present study, we apply a simplified version of super-statistical analysis to a set of full-night EEG recordings. Each 30 s epoch of these recordings has been visually scored by a sleep specialist, according to the AASM (American Academy of Sleep Medicine) rules, so that the data is categorized into four different sleep stages (REM, N1, N2, and N3) and the wake state. For each sleep-stage-labeled epoch, we compute from the single-channel recordings certain statistical hyper-parameters, such as the standard deviation (STD), the kurtosis (KUR), and the skewness (SKE) of the EEG amplitude distributions. We show that also these hyper-parameters have characteristic, stage-dependent distributions, which can be used for a simple Bayesian sleep-stage detection. Moreover, we find that the hyper-parameters are not piece-wise constant, as the traditional hypnograms would suggest. Interestingly, they show rising or falling trends also within each of the sleep stages, pointing to an underlying continuous neural process that controls human sleep.

## Results

The results presented throughout this study are based on 68 independent EEG data sets from sleeping human subjects, each recorded during a full night in the sleep lab of the University Hospital Erlangen. The signals from the three EEG channels can be analyzed, in principle, on at least three different time scales:

The shortest scale corresponds to the individual time steps *t* of the raw signals, which in our case have been recorded with a rate of 256 values per second and channel.

The medium scale is that of epochs *n*, each with a duration of 30 s, corresponding to 7680 successive EEG values per channel. A sleep stage label ${S}_{n}\in \left\{{{{{\mathrm{Wake}}}}},{{{{{\mathrm{REM}}}}}},{{{{{\mathrm{N1}}}}}},{{{{{\mathrm{N2}}}}}},{{{{{\mathrm{N3}}}}}}\right\}$ has been assigned to each of these epochs by a specialist. It is noteworthy that, for simplicity, we include Wake to the list of sleep stages.

Finally, the longest scale corresponds to sleep phases *J*, which we define as the largest non-interrupted series of subsequent epochs where the subject remains in the same sleep stage *s*. It is noteworthy that, in contrast to time steps and epochs, sleep phases are time periods with a variable duration.

**Fig. 1:**
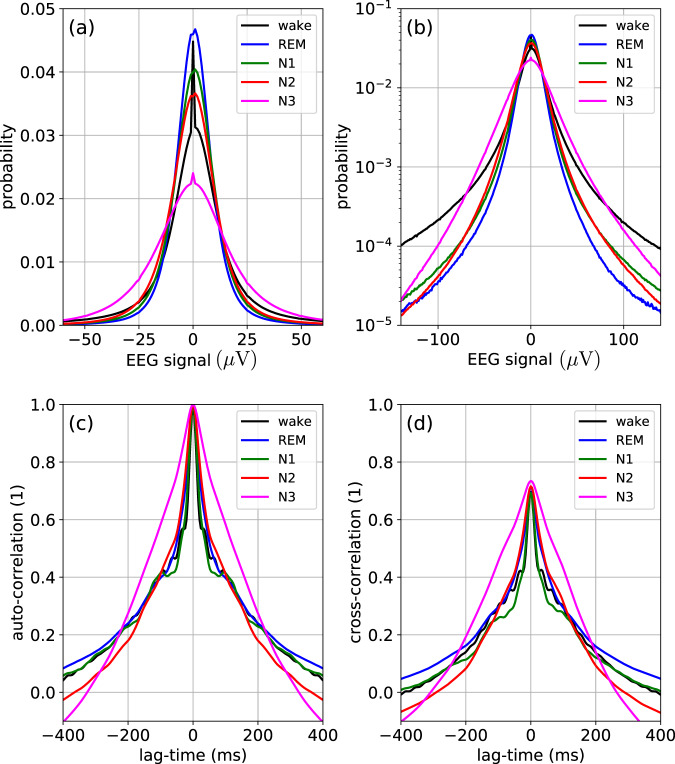
Statistical properties of raw EEG signals. Statistical properties of the raw EEG signals. **a** Linear plot of the probability density distribution. **b** Semi-logarithmic plot of the probability density distribution. **c** Autocorrelation function. **d** Cross-correlation function between channels 1 and 2.

Our first goal was to establish that single-channel EEG signals *y*_*k*_(*t*) during sleep can be considered as heterogeneous random walks, i.e., as random processes in which the statistical properties change over time, and in particular differ between sleep stages. We therefore analyze, for each sleep stage *s*, the probability distributions *p*_*s*_(*y*) of the raw EEG amplitudes (Fig. 1a, b).

The distributions are computed independently for each epoch and each channel, and finally all distributions with the same sleep stage label are pooled and averaged. We find that the amplitude distributions *p*_*s*_(*y*) are non-Gaussian and clearly leptocurtic for all sleep stages—an anomaly that is frequently found in complex systems with super-statistical parameter changes^18^. In our case, due to the rather extreme tails, the excess KUR of these distributions is unusually large (KUR_wake_ ≈ 55, KUR_REM_ ≈ 234, KUR_N1_ ≈ 154, KUR_N2_ ≈ 141, and KUR_N3_ ≈ 46). Although the distributions for sleep stages REM, N1, and N2 are relatively similar to each other, the wake and N3 stages are considerably broader, which reflects the heterogeneity of the underlying random process.

Furthermore, we compute the autocorrelation function (ACF) *A*_*n*,*k*_(Δ*t*) and the cross-correlation function *C*_*n*,1,2_(Δ*t*) between channels F4-M1 and C4-M1 (Fig. 1c, d). Here, too, the correlation functions are first computed independently for each epoch and later averaged. For all sleep stages, the EEG amplitudes show positive temporal auto-correlations up to lag times of 300 ms and very similar results are found for the cross-correlation functions. Interestingly, the N3 stage again differs from the other sleep stages, in that it has significantly stronger correlations for lag times shorter than about 200 ms.

Having thus established the heterogeneous character of the raw EEG signals during sleep, we turn to a super-statistical analysis and compute certain statistical hyper-parameters from each epoch of these raw signals. In particular, we consider as hyper-parameters the STD of the amplitude distribution *p*_*s*_(*y*), its excess KUR, its SKE, as well as the value of the ACF at specific lag time Δ*t* = 300 ms, which yields relatively large differences between the sleep stages.

**Fig. 2:**
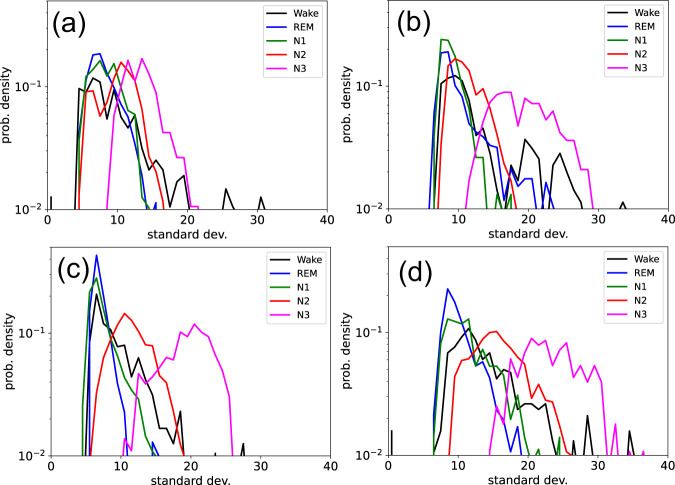
Variation of hyper-parameter distributions among subjects. Distribution of the STD (one of the hyper-parameters considered in Fig. 3 of the main paper) in the different sleep stages, plotted individually for four subjects (**a**–**d**). The characteristic differences between the stages are visible for each individual, demonstrating the temporal heterogeneity of the process. At the same time, there exist significant differences between the individuals.

**Fig. 3:**
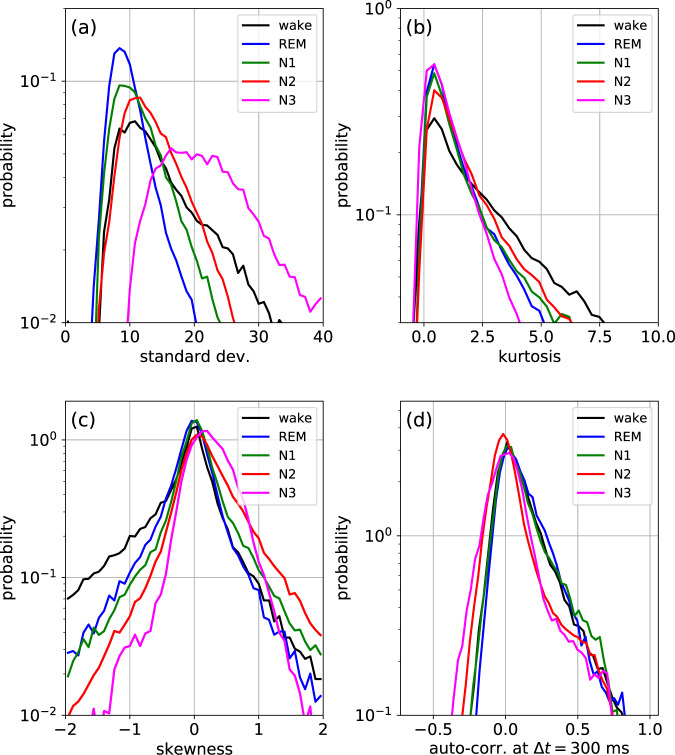
Sleep-stage-dependent distributions of selected hyper-parameters. Probability density distributions of hyper-parameters extracted from the raw EEG data shown in Fig. 1. **a** Standard deviation STD. **b** Kurtosis KUR. **c** Skewness SKE. **d** Autocorrelation at lag time 300 ms, denoted as CDT.

As a preliminary step, we compute the distributions of the STDs (STE), in the different sleep stages, for individual sleeping subjects (results for the first 4 of our 68 data sets are shown in Fig. 2). In contrast to a stationary, temporally homogeneous random walk, where the parameter STE could be regarded as fixed (apart from weak sampling fluctuations), we find in our case rather wide distributions that clearly differ between the sleep stages. The large fluctuation width of STE within the same sleep stage is pointing to dynamical changes of brain activity that are going on continuously, rather than happening only at the transition points to new sleep stages. Although there is a large degree of variability among different individuals (heterogeneity of the ensemble), the sleep-stage-specific characteristics of the distributions (temporal heterogeneity) are approximately conserved. A similar behavior is found for all considered hyper-parameters (STD, KUR, SKE, and ACF) and we therefore have pooled the data over all individuals (Fig. 3). Here we find that for some of the hyper-parameters (KUR, SKE, and ACF), the sleep-stage-specific differences are mainly visible in the tails of the distributions, correponding to the statistics of extreme values.

**Fig. 4:**
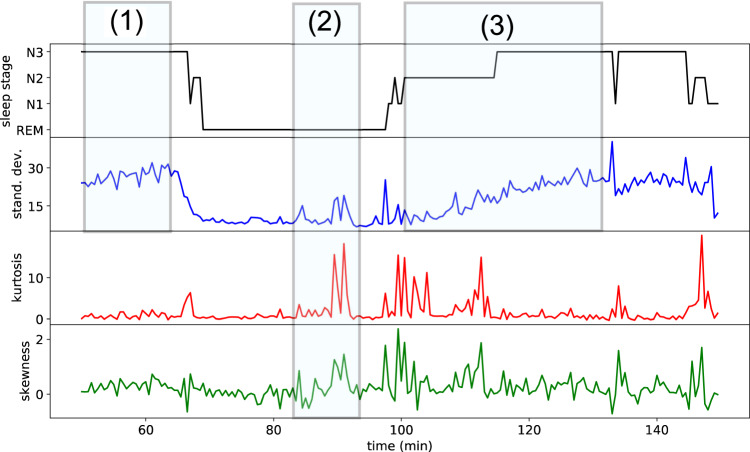
Temporal evolution of hyper-parameters and trend analysis. Typical features in the temporal behavior of hyper-parameters, such as consistent trends (1) and extreme fluctuations (2) within an ongoing sleep stage, as well as trends that extend across sleep stages (3).

In the next step, we inspect the temporal evolution of the hyper-parameters (Fig. 4). We repeatedly observe extreme bursts that exceed the normal range of fluctuations (see shaded area (2) of Fig. 4). Moreover, we frequently find that certain hyper-parameters rise or fall consistently within and also across sleep phases (see the evolution of the STD in the shaded areas (1) and (3) of Fig. 4).

In order to substantiate these anecdotal observations, we divide the 68 data sets into contiguous sleep phases, i.e., into the longest possible sequences of epochs where a subject remains within the same sleep stage. In Table 1, we further distinguish between sleep stages in the falling and rising part of the sleep cycle. In each sleep phase, the sequence of hyper-parameters is least-square-fitted by a linear function and the slope of this function, quantifying the overall trend of the hyper-parameter evolution, is extracted. Finally, we compute the mean slope and its associated error, for each of the eight sleep stages along the cycle. Although the error often exceeds 50% of the mean, we find that some hyper-parameters have clear positive or negative trends that are characteristic for each sleep stage.

**Table 1:** Average slope of hyper-parameters in different sleep stages.

Stage	Slope STD	Error	Slope Kurtosis	Error	Slope Skewness	Error
Wake	−7.905	0.699	−0.765	0.226	+0.154	0.028
REM*↓*	+1.300	0.167	+0.265	0.049	−0.017	0.008
N1*↓*	−3.351	0.256	−0.901	0.176	+0.083	0.016
N2*↓*	+0.367	0.078	−0.351	0.149	−0.017	0.012
N3	+0.861	0.318	+0.074	0.066	−0.022	0.007
N2*↑*	+2.721	0.187	+0.676	0.063	−0.042	0.009
N1*↑*	+3.010	0.689	+0.110	0.323	−0.106	0.032
REM*↑*	+0.567	0.243	+0.153	0.156	+0.014	0.024

Average slope of hyper-parameters (computed from linear fits) in different sleep stages. The stages REM, N1, and N2 have been separately evaluated for the falling (↓) and rising (↑) phases of the sleep cycle. Some hyper-parameters have clear trends, with either a positive or negative sign, depending on the sleep stage.

**Fig. 5:**
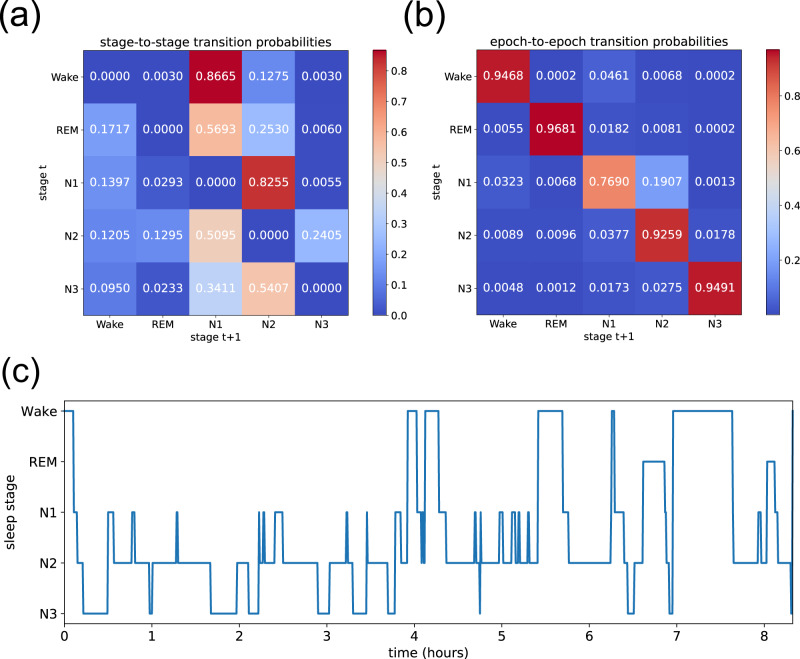
Transition probabilities between sleep stages and the simulation of hypnograms. Transition probabilities (color coded) from one non-interrupted sleep stage to the next (**a**) and from one 30 s epoch to the next (**b**). The stage-to-stage probabilities describe a strong propensity for transitions from Wake to N1 and from there to N2. After this, the most likely behavior is an oscillation between N1 and N2, or between N2 and N3. **c** Example of a simulated hypnogram, where the random walk between sleep stages is modeled as a Markov process, based on the epoch-to-epoch transition probabilities (top right).

In the next step, we consider sleep as a random walk through the discrete state space of the five sleep stages. In this context, we have computed the transition probabilities between subsequent sleep phases and between subsequent epochs, which are presented in the form of 5 × 5 transition matrices in Fig. 5a, b.

The resulting elements of the phase-to-phase transition matrix (a) show that every stage has a preferred successor stage (i.e., each row in the matrix has a single, clearly dominating entry). This leads to the emergence of a default sequence of stages: Wake → N1 → N2. After this, an ongoing oscillation N2 ↔ N1 is most probable, followed by a final descent to N3, and eventually the subject will again rise up towards the next wake state. In the case of the epoch-to-epoch transition matrix (b), all diagonal elements are close to one, reflecting the strong temporal persistence of each sleep stage.

The epoch-to-epoch transition matrix can be directly used to construct a first-order Markov model for the stochastic succession of sleep stages, as has already been demonstrated before^20^. Using such a model, an arbitrary number of simulated hypnograms can be sampled and a typical example is shown in Fig. 5c.

By comparing certain higher-level features of simulated hypnograms (such as the number of oscillations between the non-REM sleep stages) with those of actual data, it may be possible to test the validity of the first-order Markov process as a model for sleep in future work. Moreover, it may be possible to define a quantitative measure of sleep quality, based on the 25 entries of an individuals epoch-to-epoch transition matrix, compared to the corresponding values in a reference group of healthy sleepers.

The fact that each sleep stage has a specific distribution of hyper-parameters (compare again Fig. 3) does not only confirm the heterogeneous, non-stationary character of the full-night EEG signals, but it can also be exploited for an automated sleep stage detection. As a proof-of-concept, we have implemented a simple Bayesian sleep stage detector, which uses the epoch-to-epoch transition matrix as a prior and the three hyper-parameter distributions of STD, KUR, and SKE as likelihood factors.

The detector takes as input an arbitrary 30 s epoch of raw single-channel EEG signal and then computes as an output the posterior probabilities of the five sleep stages for this epoch. Although it is readily possible to extract the most probable sleep stage from these five continuous values, the distribution as a whole provides important information about the trustworthiness of the prediction, as sometimes several sleep stages can have simultaneously large probabilities.

**Fig. 6:**
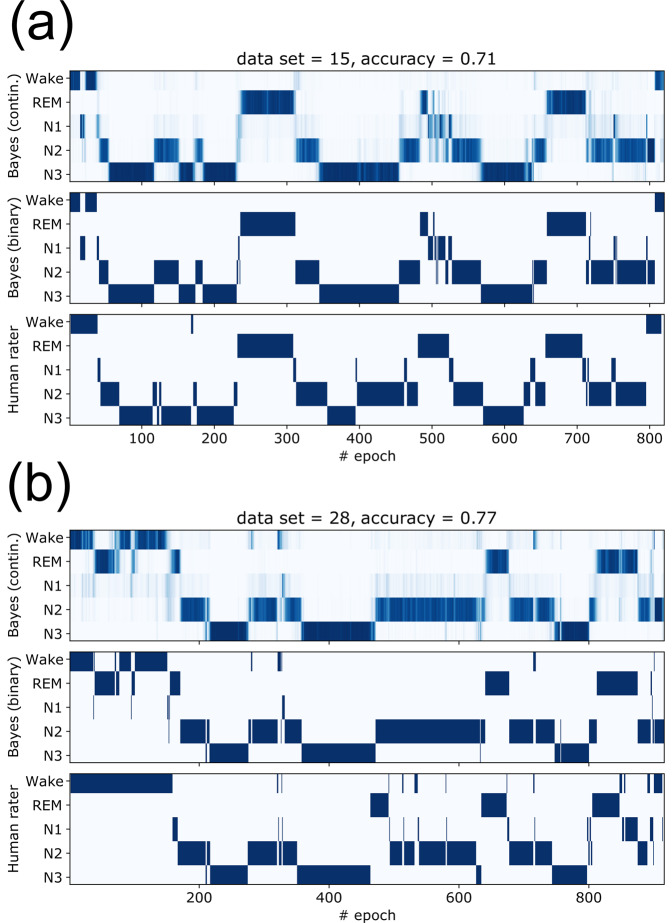
Bayesian sleep-stage classification. **a**, **b** Two examples of automated Bayesian sleep stage classification. In each case, the upper hypnogram shows, for each 30 s epoch, the posterior probabilities of the sleep stages, with larger color density corresponding to larger probability. The middle hypnogram shows only the predicted sleep stage with maximum posterior probability. The lower hypnogram is the ground truth, provided by the specialist human rater. The accuracies are defined as the ratio of correct sleep stage predictions.

Although in the present implementation the selection of hyper-parameters is arbitrary and the detector has not been optimized in any way, the predictions of the detector are in some cases very close to the ground truth of the human somnologist (Fig. 6a, b).

**Fig. 7:**
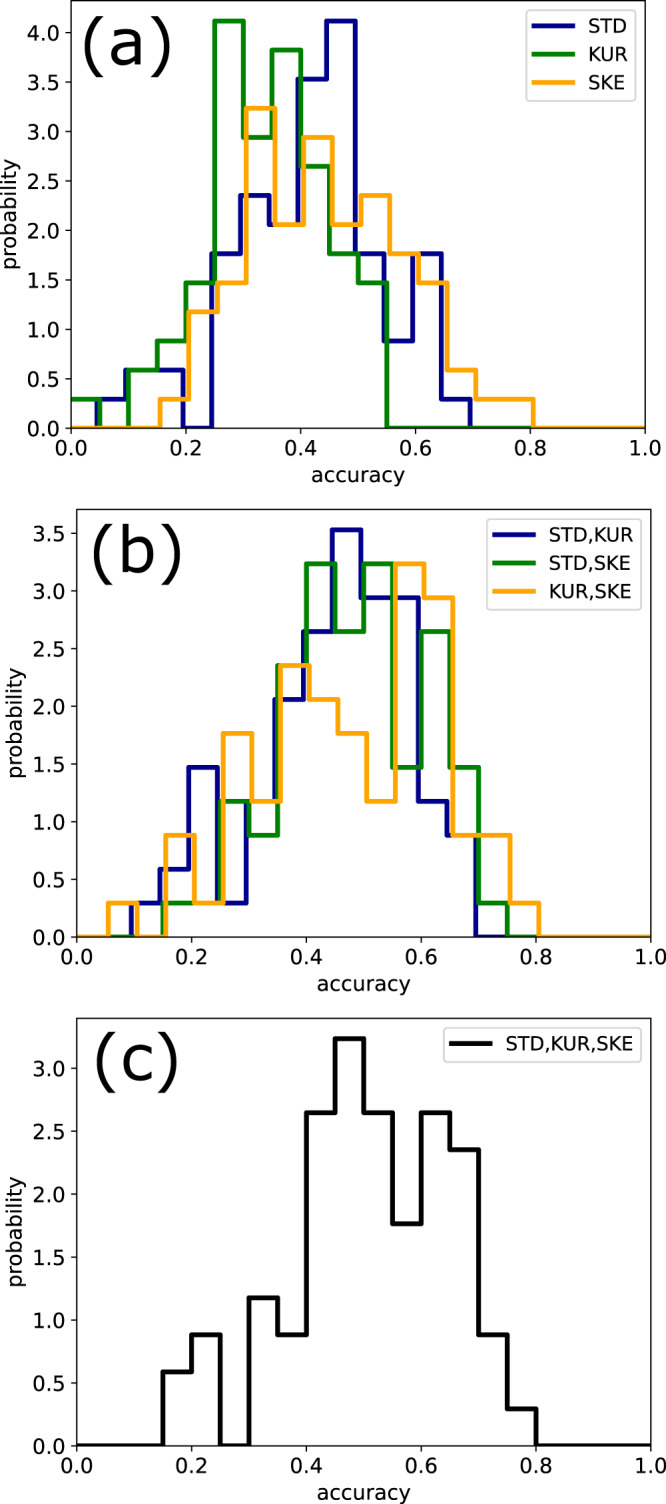
Dependence of classification accuracy on the used hyper-parameter combination. Distributions of accuracies over all 68 data sets, with different combinations of hyper-parameters used in the Bayesian likelihood: **a** Single hyper-parameters. Here, the individual mean accuracies are *μ*_STD_ = 0.42, *μ*_KUR_ = 0.34, *μ*_SKE_ = 0.44, and the global mean of these three values is *μ*_glo_ = 0.40. **b** Pairs of hyper-parameters. Here, *μ*_STD,KUR_ = 0.45, *μ*_KUR,SKE_ = 0.47, *μ*_STF,SKE_ = 0.48, and the global mean is *μ*_glo_ = 0.47. **c** All three hyper-parameters. Here the global mean is *μ*_glo_ = 0.51. The global mean is systematically increasing with the number of used hyper-parameters.

Moreover, we have computed the distribution of prediction accuracies (defined as the fraction of correctly classified epochs), including all our 68 independent data sets. The distributions systematically shift to larger values, i.e., prediction performance becomes better, when more hyper-parameters are included into the Bayesian likelihood (Fig. 7).

**Fig. 8:**
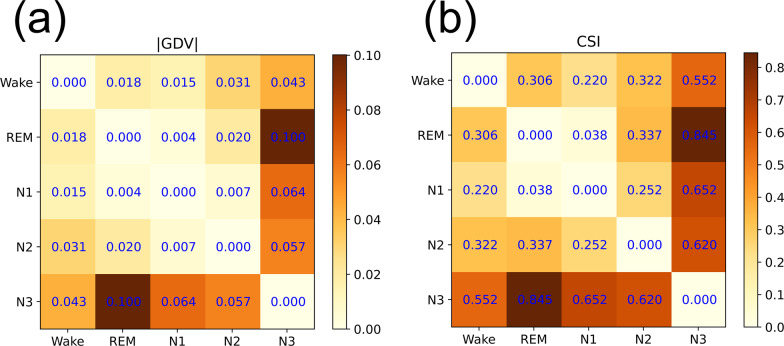
Mutual distance between sleep stages. Mutual distance between EGG data from different sleep stages, evaluated in the embedding space of the three hyper-parameters STD, KUR, and SKE. The distance matrix (**a**) shows the magnitude of the General Discrimination Value (GDV) and the matrix (**b**) shows the Cluster Separation Index (CSI). In both measures, the minimum distance is found between REM and N1, whereas the maximum distance is between REM and N3.

In our super-statistical approach, each epoch of the original EEG signals is mapped onto a small tuple of *K* hyper-parameters. This corresponds to a strong dimensionality reduction (in our case from 7680 subsequent EEG values down to only *K* = 3 hyper-parameters) and it is therefore interesting how much information can be preserved in this data compression process. Ideally, all epochs from the same sleep-state should be mapped to the same cluster of points in the *K*-dimensional embedding space and the achievable accuracy of the Bayesian detector is fundamentally limited by the degree to which these clusters overlap. We thus perform a quantitative analysis of the separability of the five sleep-stage-specific clusters in the space of the three hyper-parameters STD, KUR, and SKE. For this purpose, we use two independent measures of cluster separability, the well-established General Discrimination Value (GDV)^11^, as well as a more sophisticated measure, called the Cluster Separation Index (CSI) (cf. “Methods” section). These quantities are used to compute the pairwise distances, i.e., dissimilarities, between the sleep stages (Fig. 8). In both measures, the minimum distance is found between REM and N1, which means that these sleep stages are most similar. In contrast, the maximum distance was found between REM and N3, indicating that these sleep stages are most dissimilar to each other. These observations fit very well to the known physiology underlying the respective sleep stages. Stage N3, also called *δ*-sleep or slow-wave sleep, respectively, represents deep sleep and is physiologically characterized by highly synchronized, low-frequency, and large-amplitude cortical activity^21,22^. In contrast, both stages REM and N1 are dominated by asynchronous, low-amplitude, and high-frequency cortical activity^21,22^. These sleep stages resemble wakefulness and thus represent the physiological opposite of N3.

## Discussion

Traditionally, the analysis of EEG recordings has mainly focused on the oscillatory features of the signals, such as *α*-, *β*-, *δ*-, and *θ*-frequency bands, and in the context of sleep also on wavelet-like features, i.e., grapho-elements, such as sleep spindles or K-complexes. Just recently, it became clear that also the aperiodic component of an EEG signal, in particular the ubiquitous background noise with a *f*^−*β*^-like power spectrum, contains valuable information about the physiological state of the subject^23,24^. Indeed, these aperiodic, scale-free fluctuations have been shown to systematically change with age and with the tasks to be performed^25^. Moreover, they offer a novel way to asses the level of arousal^26^.

An alternative approach is to focus neither on oscillatory features, nor on the global power spectrum, but to treat each single-channel EEG signal simply as a random walk. As has been found very early on^27,28^, the changes between two subsequent EEG values (the steps of the walk) are not always normally distributed, and later studies have revealed further anomalous properties of EEG random walks^29–31^.

In this work, we treat the signal as a non-stationary, heterogeneous random walk, generated by a stochastic system with parameters that change over time, depending on the physiological state of the subject. In particular, this random walk has different statistical properties in each of the five sleep (or, more precisely, vigilance) stages and these differences can be exploited for a simple automated Bayesian sleep stage detection.

Although several methods of automatic sleep-stage detection are already available^32–34^, we have implemented, as a proof-of-concept, a first version of a Bayesian, hyper-parameter-based detector.

In contrast to sleep-stage detectors based on deep neural networks, which suffer from the black box problem^35^, our Bayesian approach is completely transparent and explainable, as the features used to distinguish between sleep stages (i.e., the distributions of hyper-parameters) are explicit. Once these hyper-parameter distributions are extracted from the raw data and included into the likelihood, the Bayesian detector can immediately be applied without any training or further optimization. In contrast, most deep learning applications require extensive training and are data hungry^36^. Furthermore, although the posterior probabilities of the momentary sleep stages are mathematically well-defined in the Bayesian approach, it is not clear if the typical softmax outputs of a deep neural network can actually be interpreted as probabilities, or if they are just a list of scores that sum up to one. Finally, we have shown that the accuracy of the Bayesian sleep stage detector can be systematically improved, simply by including additional hyper-parameter distributions as factors in the likelihood. In principle, the number of these factors could be made arbitrarily large by using hyper-parameters such as ${\left\langle {({y}_{t}-\overline{y})}^{m}\right\rangle }_{t\in n}$, the *m*-th central moments of the fluctuating EEG signal *y*_*t*_ within each 30 s epoch *n*, or ${\left|{\left\langle {y}_{t}{e}^{i{\omega }_{k}t}\right\rangle }_{t\in n}\right|}^{2}$, the magnitude squared of momentary Fourier components for different frequencies *ω*_*k*_.

Besides the probability distributions of the hyper-parameters, we have also studied their gradual evolution over time. Some of the hyper-parameters show consistent rising or falling trends within and across the phases in which the subject is scored to be in a constant sleep stage. For example, we have observed a case where the STD of the EEG signal is continuously increasing for about 30 min, whereas the subject is passing from the REM state through N1, to N2, and finally to the N3 state (compare Fig. 4(3)). Such a gradual buildup of EEG amplitude points to a continuous mechanism in the brain that is regulating the sleep cycle, a phenomenon similar to the change of hyper-parameters that we have observed in migrating cells during the cell cycle^18^.

We speculate that, rather than sub-dividing sleep into discrete stages, it might be useful to introduce a continuous master variable $\phi (t)\in \left[0,2\pi \right]$, roughly resembling the mathematical phase of a sinusoidal oscillation, which tends to increase about linearly with time and which reflects the momentary position of the subject within the sleep cycle. In principle, it may then be possible to design a simple stochastic first-level model, such as an auto-regressive process of low order, with coefficients that are not constant but which are top-down controlled (from a second model level) by the master variable *ϕ*(*t*). As we have demonstrated in other contexts^18,19^, such super-statistical two-level models are often capable to reproduce the anomalous time-dependent statistics of biological and other complex systems (typically involving non-normally distributed, long-time-correlated signals) in a particular simple way. In future work, one could therefore attempt to reproduce the sleep-stage-dependent properties of the EEG raw signals (Fig. 1) and of the various hyper-parameters (Fig. 3) with such a two-level model.

Indeed, it may even be possible to relate the phase variable *ϕ*(*t*) to existing models of sleep^37–41^. An obvious candidate would be the famous two-process model^42,43^, in which sleep is controlled by the nonlinear interplay between the circadian propensity for sleep, governed by an intrinsic circadian oscillator, and a homeostatic drive for sleep that continuously increases during the waking state and dissipates during sleep. In this case, the circadian and homeostatic signals may directly represent the second-level control signals of a two-level super-statistical model.

## Methods

### Heterogeneous random walks and superstatistics

In this work, the term superstatistics does not refer to the superposition of different statistical models, as originally studied by Beck and Cohen^15–17^, but more specifically to a method for the analysis of heterogeneous, time-discrete random walks, as first introduced in refs. ^18,19^. We define random walks in the broadest sense as time series of momentary values *y*(*t* = 0, 1, 2, …), which are assumed to be generated by a stochastic process. In particular, the discrete steps Δ*y*(*t*) = *y*(*t*) − *y*(*t* − 1) of such a general random walk need not to be normally distributed and there may exist linear correlations between subsequent momentary values (correlated random walk) or even more complex dependencies between values many time points appart. Moreover, the underlying stochastic process may also have some deterministic components.

A random walk is called heterogeneous if its statistical properties (such as the distribution of steps *p*(Δ*y*) or the degree of temporal correlations) change over time. As has been shown in refs. ^18,19^ and elsewhere, this can lead to anomaleous statistical properties of the random walk as a whole (such as non-Gaussian, fat-tailed step distributions *p*(Δ*y*), or long-time correlations that can be approximated by powerlaw autocorrelation functions), although each sufficiently small time interval can be described by a regular random walk (often even with Gaussian step distributions and approximately constant statistical parameters).

The method of super-statistical analysis, in its simplest implementation, therefore sub-divides the random walk into small non-overlapping time intervals (windows) and computes relevant statistical parameters (such as the *n*th-order moments of the momentary distribution function *p*(*y*)) independently for each of these time windows. In the case of a heterogeneous random walk, the resulting statistical parameters will fluctuate around their mean values much more strongly than expected from sampling statistics only. The fluctuations of the parameters can be described by (super-statistical) distribution functions, which represent characteristic properties of the heterogeneous random walk. We therefore refer to such strongly fluctuating parameters as hyper-parameters.

### Generation of data sets

This work is based on 68 independent data sets, each containing 1 full-night 3-channel EEG recording (channels F4-M1, C4-M1, and O2-M1) from a different human subject during sleep, recorded with a sampling rate of 256 Hz. For most of the following analysis, each of the three channels was treated as a different (sub-)data set and evaluated separately, except for the computation of the cross-correlation functions (see below). The participants of the study included 46 males and 22 females, with an age range between 21 and 80 years. Exclusion criteria were a positive history of misuse of sedatives, alcohol or addictive drugs, as well as untreated sleep disorders. The study was conducted in the Department of Otorhinolaryngology, Head Neck Surgery, of the Friedrich-Alexander University Erlangen-Nürnberg, following approval by the local Ethics Committee (323-16 Bc). Written informed consent was obtained from the participants before the cardiorespiratory polysomnography. After recording, the raw EEG data were analyzed by a sleep specialist accredited by the German Sleep Society, who removed typical artifacts^44^ from the data and visually identified the sleep stages in subsequent 30 s epochs, according to the AASM criteria (Version 2.1, 2014)^45,46^. The resulting, labeled raw data were then used for our standard statistical and super-statistical analysis, and also as a ground truth to test the performance of the Bayesian sleep-stage classification.

### Sleep-stage-specific statistical properties of raw EEG data

In a first step, each individual epoch *n* and channel *k* was statistically analyzed by computing the probability density distribution *p*_*n*,*k*_(*y*) of the momentary EEG signal amplitudes *y*_*n*,*k*_(*t*), their temporal ACF

1 $${A}_{n,k}({{\Delta }}t)=\frac{\left\langle \left({y}_{n,k}(t)-{\overline{y}}_{n,k}\right)\cdot \left({y}_{n,k}(t\,+\,{{\Delta }}t)-{\overline{y}}_{n,k}\right)\right\rangle }{{\sigma }_{n,k}^{2}},$$

as well as the cross-correlation function between channels 1 and 2

2 $${C}_{n,1,2}({{\Delta }}t)=\frac{\left\langle \left({y}_{n,1}(t)-{\overline{y}}_{n,1}\right)\cdot \left({y}_{n,2}(t\,+\,{{\Delta }}t)-{\overline{y}}_{n,2}\right)\right\rangle }{{\sigma }_{n,1}\cdot {\sigma }_{n,2}},$$

where ${\overline{y}}_{n,k}={\left\langle {y}_{n,k}(t)\right\rangle }_{t}$ is the temporal average of channel *k*s amplitude *y*_*n*,*k*_(*t*) within epoch *n* and ${\sigma }_{n,k}=\sqrt{{\left\langle {\left({y}_{n,k}(t)-{\overline{y}}_{n,k}\right)}^{2}\right\rangle }_{t}}$ is the corresponding STD. It is noteworthy that in this case, the STD is equivalent to the root-mean-squared amplitude values that we used in previous studies^8,9^.

In a second step, we have pooled and averaged *p*_*n*,*k*_(*y*), *A*_*n*,*k*_(Δ*t*), and *C*_*n*,1,2_(Δ*t*) over all epochs that belong to the same sleep stage *s*. The quantities *p*_*n*,*k*_(*y*) and *A*_*n*,*k*_(Δ*t*) were additionally pooled and averaged over all channels *k*. As a result, we obtain the statistical properties *p*_*s*_(*y*), *A*_*s*_(Δ*t*), and *C*_*s*,1,2_(Δ*t*) that are characteristic for each sleep stage *s* and which are shown in Fig. 1.

### Extraction and statistical analysis of hyper-parameters

Based on the raw data *y*_*n*,*k*_(*t*), we have computed for each channel *k* and epoch *n* a set of hyper-parameters, namely the STD

3 $${{{\mbox{STD}}}}_{n,k}=\sqrt{{\left\langle {\left({y}_{n,k}(t)-{\overline{y}}_{n,k}\right)}^{2}\right\rangle }_{t}},$$

the excess curtosis

4 $${{{\mbox{KUR}}}}_{n,k}={\left\langle {\left(\frac{{y}_{n,k}(t)-{\overline{y}}_{n,k}}{{\sigma }_{n,k}}\right)}^{4}\right\rangle }_{t}-3,$$

the SKE

5 $${{{\mbox{SKE}}}}_{n,k}={\left\langle {\left(\frac{{y}_{n,k}(t)-{\overline{y}}_{n,k}}{{\sigma }_{n,k}}\right)}^{3}\right\rangle }_{t}$$

and the value of the ACF at the specific lag time of 300 ms, where ACF differences between the sleep stages are relatively large:

6 $${{{\mbox{ACF}}}}_{n,k}={A}_{n,k}({{\Delta }}t\,=\,300\,{{{{{\mathrm{ms}}}}}}).$$

As these hyper-parameters are strongly fluctuating themselves, we have pooled them over all epochs and channels and computed their sleep-stage specific distribution functions *p*_*s*_(STD), *p*_*s*_(KUR), *p*_*s*_(SKE), and *p*_*s*_(ACF), which are shown in Fig. 3.

### Temporal trend analysis of hyper-parameters

For a temporal trend analysis of the hyper-parameters, we no longer partition the EEG time series *y*_*n*,*k*_(*t*) into 30 s epochs, but into longer, contiguous sleep phases: within a given full-night recording, the sleep phases *J* are defined as the longest possible continuous time periods $\left[{T}_{J,{{{{{\mathrm{beg}}}}}}},{T}_{J,{{{{{\mathrm{end}}}}}}}\right]$, in which the subject was scored to be in the same constant sleep stage *s* = *s*(*J*). Typically, each sleep phase *J* contains a large number of subsequent epochs *n*. The hyper-parameters STD_*n*,*k*_, KUR_*n*,*k*_, … perform a higher-order random walk within each sleep phase *J*, and visual inspection reveals that some of these random walks have rising and falling trends (Fig. 4).

To evaluate these trends, we approximate the time series of the hyper-parameters within each contiguous sleep phase by a linear function, *f*_*hyp*,*J*_(*n*) ≈ *a*_*J*_ × *n* + *b*_*J*_, using least-square fits. The slopes *a*_*J*_ of these linear fits are then pooled and averaged over all sleep phases *J* with the same sleep stage *s*. The results are shown in Table 1. It is noteworthy that here we have sub-divided the sleep stages REM, N1, and N2 into the falling and the rising part of the oscillatory motion between the two extreme stages of Wake and N3.

### Evaluation of transition probabilities between sleep stages

The sequence of human-scored sleep stage labels *s*_*n*_ for each subsequent epoch *n* can be regarded as a random walk in a discrete state space ${s}_{n}\in \left\{{{{{{\mathrm{Wake}}}}}},{{{{{\mathrm{REM}}}}}},{{{{{\mathrm{N1}}}}}},{{{{{\mathrm{N2}}}}}},{{{{{\mathrm{N3}}}}}}\right\}$. As this discrete random walk shows clear temporal correlations, we have evaluated the (normalized) transition probabilities *p*(*s*_*J*+1_∣*s*_*J*_) between subsequent sleep phases, as well as the transition probabilities *p*(*s*_*n*+1_∣*s*_*n*_) between subsequent epochs. The resulting transition matrices are shown in Fig. 5a, b. It is worth noting that, by definition, the diagonal elements of the phase-to-phase transition matrix are zero. By contrast, the diagonal elements of the epoch-to-epoch transition matrix are relatively close to one, as each sleep stage has a high degree of persistence.

The epoch-to-epoch transition matrix defines a Markov random process of first order. After defining the starting stage *s*_*n*=0_, the transition matrix can be used to simulate an arbitrarily long sequence of sleep stages. An example is shown in the hypnogram of Fig. 5c.

### Bayesian sleep stage prediction

We have implemented a simple Bayesian model that predicts the probabilities *P*(*s*_*n*_) of the sleep labels ${s}_{n}\in \left\{{{{{{\mathrm{Wake}}}}}},{{{{{\mathrm{REM}}}}}},{{{{{\mathrm{N1}}}}}},{{{{{\mathrm{N2}}}}}},{{{{{\mathrm{N3}}}}}}\right\}$ from the raw EEG data *D*_*n*_ in each 30 s epoch *n* (note that *D*_*n*_ here stands for the complete set of 30 × 256 successive EEG values corresponding to the given epoch *n*). The prediction is based on the momentary values *h*_*kn*_ of selected statistical hyper-parameters (in our case, the STD *h*_1*n*_, the KUR *h*_2*n*_, and the SKE *h*_3*n*_), which are calculated directly from the data *D*_*n*_, and which have different likelihoods *q*(*h*_*kn*_∣*s*_*n*_) in the various sleep stages *s*_*n*_. Furthermore, we take into account the prior probability Π(*s*_*n*_) of the momentary sleep stage, which depends on the prediction *P*(*s*_*n*−1_) from the last epoch and on the known transition probability *M*(*s*_*n*_∣*s*_*n*−1_). The prediction for the current epoch is then given by

7 $$P({s}_{n})=\frac{Q({D}_{n}| {s}_{n})\cdot {{\Pi }}({s}_{n})}{{\sum }_{{s}_{n}^{\prime}}Q({D}_{n}| {s}_{n}^{\prime})\cdot {{\Pi }}({s}_{n}^{\prime})}\ .$$

Here, the global likelihood *Q*(*D*_*n*_∣*s*_*n*_) of the current data epoch *D*_*n*_ is given as the product over the individual likelihoods of the different hyper-parameters *h*_*kn*_:

8 $$Q({D}_{n}| {s}_{n})=\mathop{\prod}\limits_{k={{{{{\mathrm{STD}}}}}},\ldots }q({h}_{kn}| {s}_{n})=q({h}_{{{{{{\mathrm{STD}}}}}},n}| {s}_{n})\cdot q({h}_{{{{{{\mathrm{KUR}}}}}},n}| {s}_{n})\cdot \ldots \ .$$

We have numerically implemented these likelihood distribution as continuous spline-extrapolations that were pre-computed from empirical histograms with discrete bins. In this way, also new data can be handled with extreme values of the hyper-parameters that are outside of the empirical histograms. Another possible implementation would be via kernel density distributions. The (normalized) prior probability is computed as

9 $${{\Pi }}({s}_{n})=\frac{\ \ \ \ \ \ {\sum }_{{s}_{n-1}}M({s}_{n}| {s}_{n-1})\cdot P({s}_{n-1})}{{\sum }_{{s}_{n}^{\prime}}{\sum }_{{s}_{n-1}}M({s}_{n}^{\prime}| {s}_{n-1})\cdot P({s}_{n-1})}.$$

In the initial epoch *n*_0_, we assume for simplicity that the subject is in the wake state. For occasional epochs in which the raw EEG data are not reliable due to obvious measurement artifacts, Bayesian updating proceeds only on the basis of the prior Π.

### Separability of sleep stages

The accuracy of automatic sleep-stage detection depends on how well data clusters from different stages separate in the embedding space, which in our case corresponds to the three-dimensional space of the hyper-parameters STD, KUR, and SKE. In order to assess this mutual separability of sleep stages in a quantitative way, we use two related measures: the GDV^8,9,11^ and the CSI. Both measures take as an input a list of *N* labeled *D*-dimensional data vectors (points), belonging to *L* distinct classes (clusters) and produce as an output a single number that characterizes the degree of separability of these classes. Also, both measures consider two classes as well, separable if the Euclidean distance of data points between the two classes is typically much larger than the distance of points within the same class.

#### Generalized discrimination value

We consider *N* points **x**_**n=1..N**_ = (*x*_*n*,1_, ⋯ , *x*_*n*,*D*_), distributed within *D*-dimensional space. A label *l*_*n*_ assigns each point to one of *L* distinct classes *C*_*l*=1..*L*_. In order to become invariant against scaling and translation, each dimension is separately *z*-scored and, for later convenience, multiplied with $\frac{1}{2}$:

10 $${s}_{n,d}=\frac{1}{2}\cdot \frac{{x}_{n,d}-{\mu }_{d}}{{\sigma }_{d}}.$$

Here, ${\mu }_{d}=\frac{1}{N}{\sum }_{n = 1}^{N}{x}_{n,d}$ denotes the mean and ${\sigma }_{d}=\sqrt{\frac{1}{N}{\sum}_{n = 1}^{N}{({x}_{n,d}-{\mu}_{d})}^{2}}$ the STD of dimension *d*. Based on the re-scaled data points **s**_**n**_ = (*s*_*n*,1_, ⋯ , *s*_*n*,*D*_), we calculate the mean intra-class distances for each class *C*_*l*_

11 $$\bar{d}({C}_{l})=\frac{2}{{N}_{l}({N}_{l}\,-\,1)}\mathop{\sum }\limits_{i=1}^{{N}_{l}-1}\mathop{\sum }\limits_{j=i+1}^{{N}_{l}}d({{{{{{{{\bf{s}}}}}}}}}_{i}^{(l)},{{{{{{{{\bf{s}}}}}}}}}_{j}^{(l)}),$$

and the mean inter-class distances for each pair of classes *C*_*l*_ and *C*_*m*_

12 $$\bar{d}({C}_{l},{C}_{m})=\frac{1}{{N}_{l}{N}_{m}}\mathop{\sum }\limits_{i=1}^{{N}_{l}}\mathop{\sum }\limits_{j=1}^{{N}_{m}}d({{{{{{{{\bf{s}}}}}}}}}_{i}^{(l)},{{{{{{{{\bf{s}}}}}}}}}_{j}^{(m)}).$$

Here, *N*_*k*_ is the number of points in class *k* and ${{{{{{{{\bf{s}}}}}}}}}_{i}^{(k)}$ is the *i*th point of class *k*. The quantity *d*(**a**, **b**) is the euklidean distance between **a** and **b**. Finally, the GDV is calculated from the mean intra-class and inter-class distances as follows:

13 $$\,{{\mbox{GDV}}}\,=\frac{1}{\sqrt{D}}\left[\frac{1}{L}\mathop{\sum }\limits_{l=1}^{L}\bar{d}({C}_{l})\ -\ \frac{2}{L(L\,-\,1)}\mathop{\sum }\limits_{l = 1}^{L-1}\mathop{\sum }\limits_{m=l+1}^{L}\bar{d}({C}_{l},{C}_{m})\right]$$

whereas the factor $\frac{1}{\sqrt{D}}$ is introduced for dimensionality invariance of the GDV with *D* as the number of dimensions. In the case of two Gaussian distributed point clusters, the resulting discrimination value becomes −1.0 if the clusters are located such that the mean inter cluster distance is two times the STD of the clusters.

#### Cluster separation index

The basic idea of the GDV is to compare the distance between two clusters with the size of each individual cluster. However, as cluster size is computed as an average over all point-to-point distances, this quantity can become relatively large in highly non-spherical clusters, e.g., when the points are distributed linearly along a straight or curved line. For this reason, the GDV may consider two parallel, line-like clusters 1 and 2 as not well separated, even if each point in 1 is much closer to some adjacent point of 1 than to any point in 2. To resolve this problem, we have defined an alternative measure of class separability, the CSI, which is based on nearest-neighbor distance relations and which resembles a quantity used before for margin-based feature selection^47^.

In order to determine the CSI of a labeled set of *N* data points in *D*-dimensional space, we compute for each data point *n* the Euclidean distance ${d}_{n}^{\ ({{{{{\mathrm{min}}}}}},S)}$ to its nearest neighbor within the same class, as well as the distance ${d}_{n}^{\ ({{{{{\mathrm{min}}}}}},O)}$ to its nearest neighbor among all the other classes. The CSI is then defined as the logarithm of the ratio of these two distances, averaged over all data points in all classes:

14 $$\,{{\mbox{CSI}}}\,={\left\langle {{{{{{\mathrm{ln}}}}}}}\,\left({d}_{n}^{({{{{{\mathrm{min}}}}}},O)}/{d}_{n}^{({{{{{\mathrm{min}}}}}},S)}\right)\right\rangle }_{n}$$

Here, it is assumed that all point-to-point distances in the data are non-zero. As the CSI is based on the ratio of Euclidean distances, it is invariant against translation and scaling. According to the CSI, two parallel line-like clusters are considered as well separated, provided that the density of points within each line is sufficiently large.

It is noteworthy that both the GDV and the CSI produce values around zero when clusters are not separable. However, as separability increases, the GDV becomes more negative and the CSI more positive. To make both measures better comparable, we are considering the magnitude ∣*GDV*∣ in Fig. 8, where the mutual distances between hyper-parameter clusters from different sleep stages are presented.

### Reporting summary

Further information on research design is available in the Nature Research Reporting Summary linked to this article.

## Supplementary information

Description of Additional Supplementary Files

Supplementary Software 1

Supplementary Data 1

Reporting Summary

## Data Availability

In the online repository figshare (10.6084/m9.figshare.17113664), we provide all required data (with associated Python 3.9.5 scripts) to reproduce the 8 figures of the paper (“Supplementary Data 1.zip”).

## Code availability

In the online repository figshare (10.6084/m9.figshare.17113700), we provide a Python 3.9.5 program (including test data) to run the Bayesian sleep stage detector (“Supplementary Software 1.zip”).
