# Supplementary material for: Sleep as a random walk: a super-statistical analysis of EEG data across sleep stages
Source: Commun Biol. 2021 Dec 10;4:1385. doi: 10.1038/s42003-021-02912-6 (PMC8664947; doi:10.1038/s42003-021-02912-6)
Supplement: Supplementary file 1 — Description of Additional Supplementary Files [file 42003_2021_2912_MOESM1_ESM.pdf]

Description of Additional Supplementary Files

File name: Supplementary Software 1.
Description: Python 3.9.5 implementation (including test data) of the Bayesian sleep stage detector.

File name: Supplementary Data 1.
Description: Required data (with associated Python 3.9.5 scripts) to reproduce the 8 figures of the paper.
