# Supplementary figures and images for: Sleep as a random walk: a super-statistical analysis of EEG data across sleep stages
Source: Commun Biol. 2021 Dec 10;4:1385. doi: 10.1038/s42003-021-02912-6 (PMC8664947; doi:10.1038/s42003-021-02912-6)

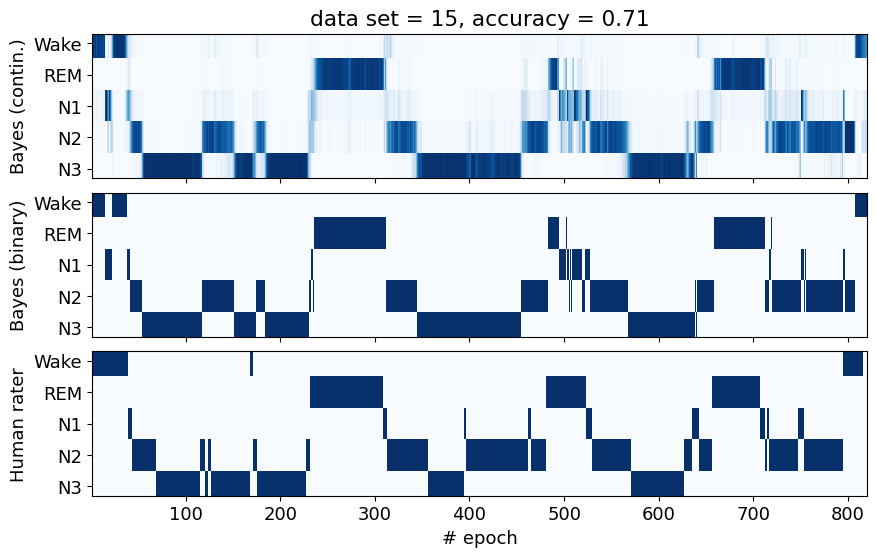

Supplement: Supplementary file 2 — Supplementary Software 1 [file 42003_2021_2912_MOESM2_ESM.zip › BayesHypnoTracker1/hypnograms/hypnogram_15.png]

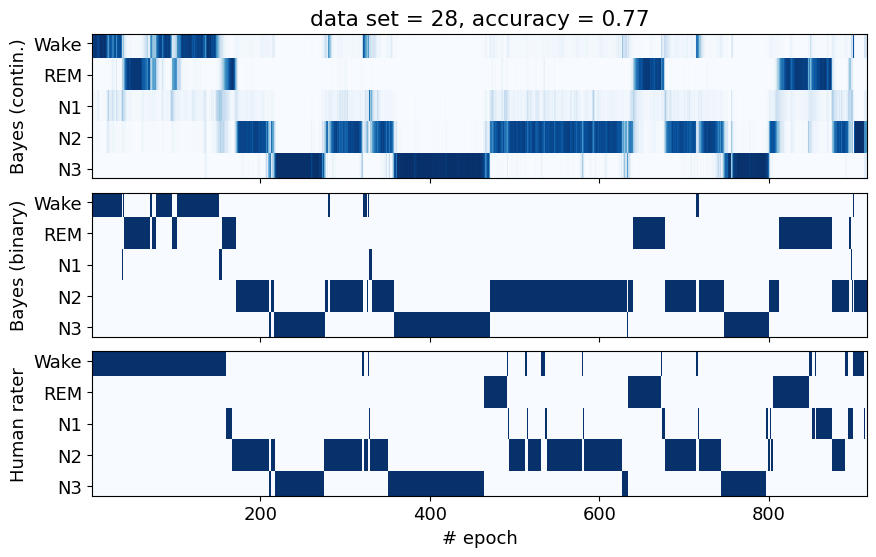

Supplement: Supplementary file 2 — Supplementary Software 1 [file 42003_2021_2912_MOESM2_ESM.zip › BayesHypnoTracker1/hypnograms/hypnogram_28.png]

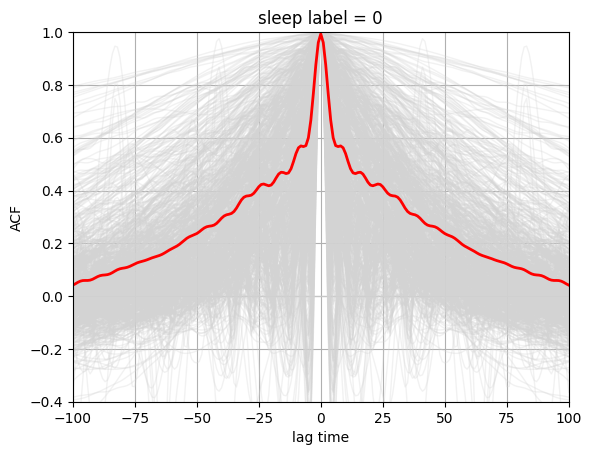

Supplement: Supplementary file 3 — Supplementary Data 1 [file 42003_2021_2912_MOESM3_ESM.zip › SupplementaryData1/Prg_Figure1/ACF/Acc_stage_0.png]

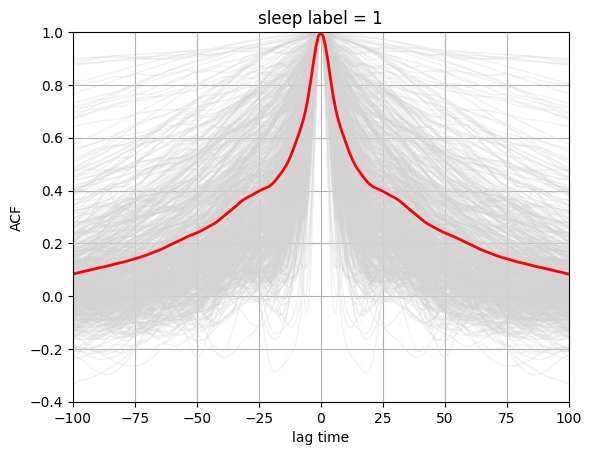

Supplement: Supplementary file 3 — Supplementary Data 1 [file 42003_2021_2912_MOESM3_ESM.zip › SupplementaryData1/Prg_Figure1/ACF/Acc_stage_1.png]

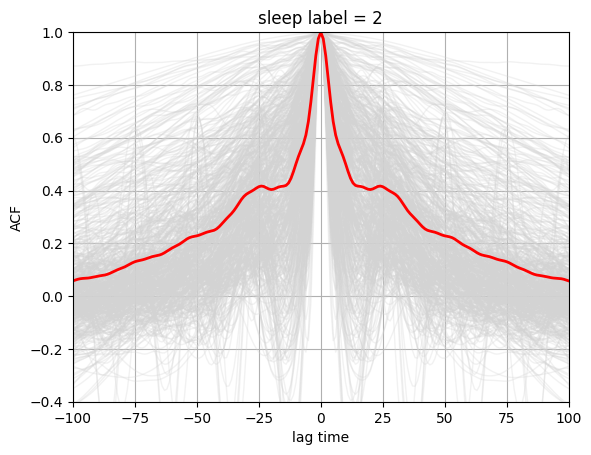

Supplement: Supplementary file 3 — Supplementary Data 1 [file 42003_2021_2912_MOESM3_ESM.zip › SupplementaryData1/Prg_Figure1/ACF/Acc_stage_2.png]

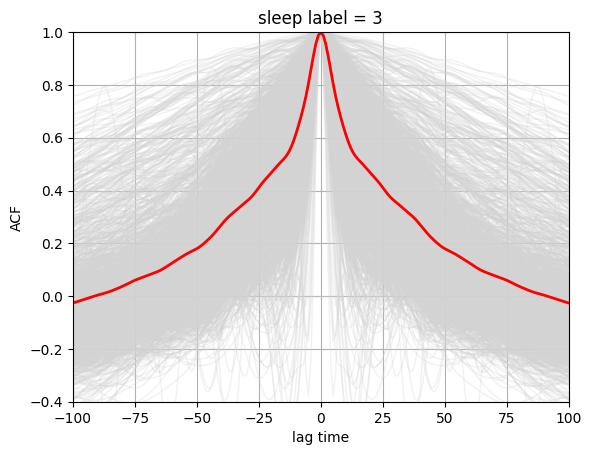

Supplement: Supplementary file 3 — Supplementary Data 1 [file 42003_2021_2912_MOESM3_ESM.zip › SupplementaryData1/Prg_Figure1/ACF/Acc_stage_3.png]

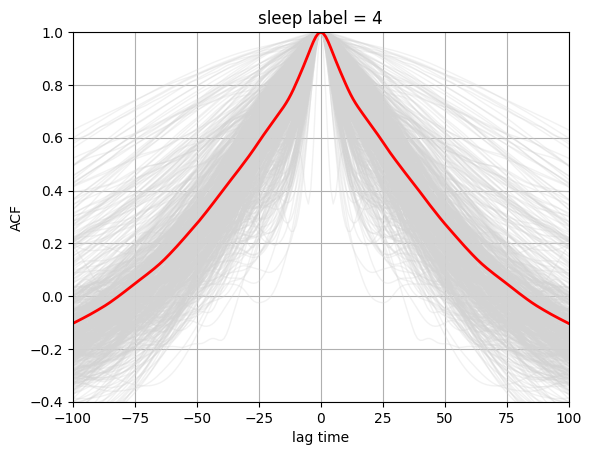

Supplement: Supplementary file 3 — Supplementary Data 1 [file 42003_2021_2912_MOESM3_ESM.zip › SupplementaryData1/Prg_Figure1/ACF/Acc_stage_4.png]

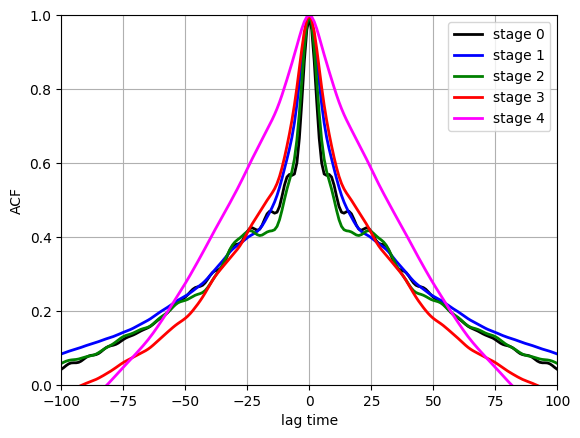

Supplement: Supplementary file 3 — Supplementary Data 1 [file 42003_2021_2912_MOESM3_ESM.zip › SupplementaryData1/Prg_Figure1/ACF/AverageACFs.png]

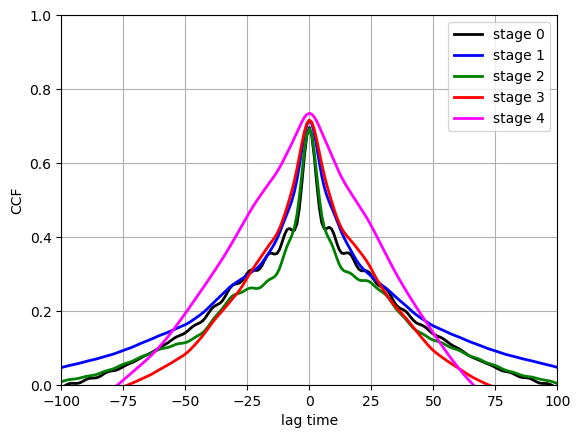

Supplement: Supplementary file 3 — Supplementary Data 1 [file 42003_2021_2912_MOESM3_ESM.zip › SupplementaryData1/Prg_Figure1/CCF/AverageCCFs.png]

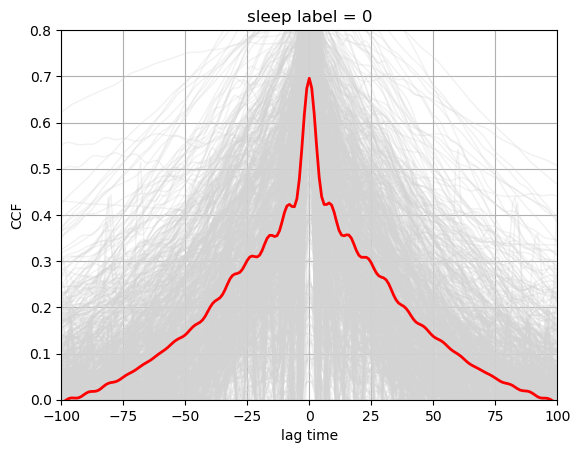

Supplement: Supplementary file 3 — Supplementary Data 1 [file 42003_2021_2912_MOESM3_ESM.zip › SupplementaryData1/Prg_Figure1/CCF/CCF_stage_0.png]

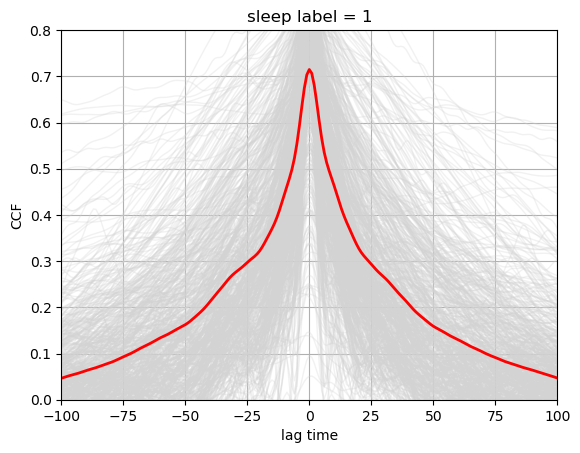

Supplement: Supplementary file 3 — Supplementary Data 1 [file 42003_2021_2912_MOESM3_ESM.zip › SupplementaryData1/Prg_Figure1/CCF/CCF_stage_1.png]

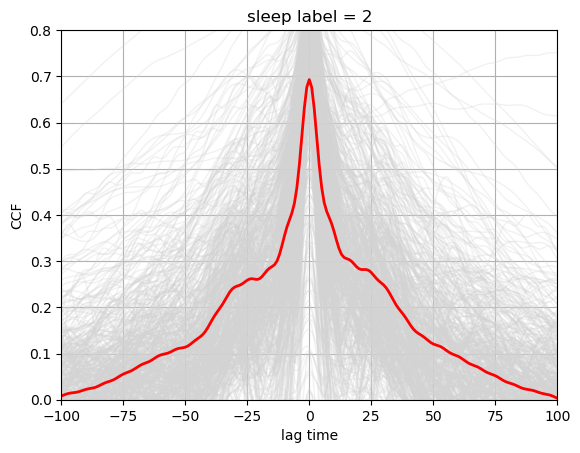

Supplement: Supplementary file 3 — Supplementary Data 1 [file 42003_2021_2912_MOESM3_ESM.zip › SupplementaryData1/Prg_Figure1/CCF/CCF_stage_2.png]

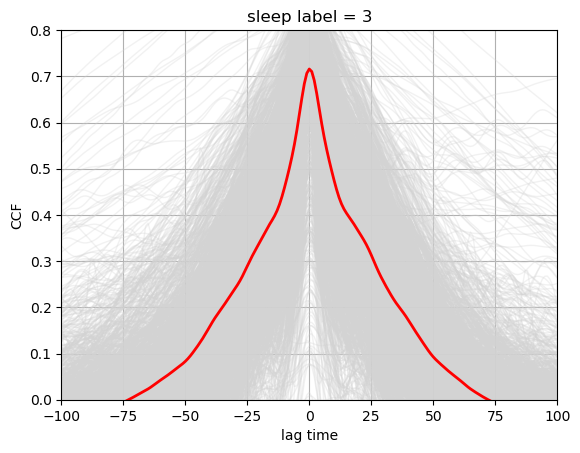

Supplement: Supplementary file 3 — Supplementary Data 1 [file 42003_2021_2912_MOESM3_ESM.zip › SupplementaryData1/Prg_Figure1/CCF/CCF_stage_3.png]

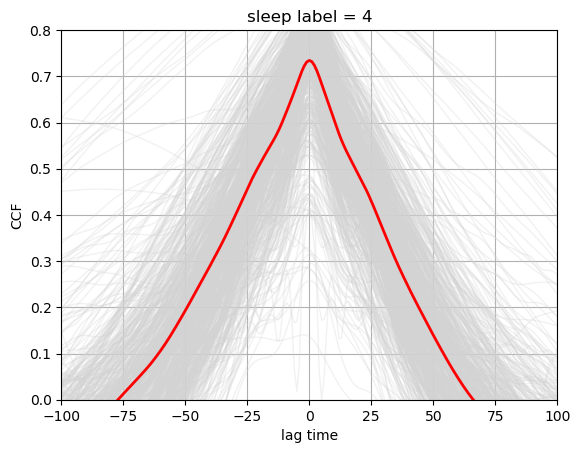

Supplement: Supplementary file 3 — Supplementary Data 1 [file 42003_2021_2912_MOESM3_ESM.zip › SupplementaryData1/Prg_Figure1/CCF/CCF_stage_4.png]

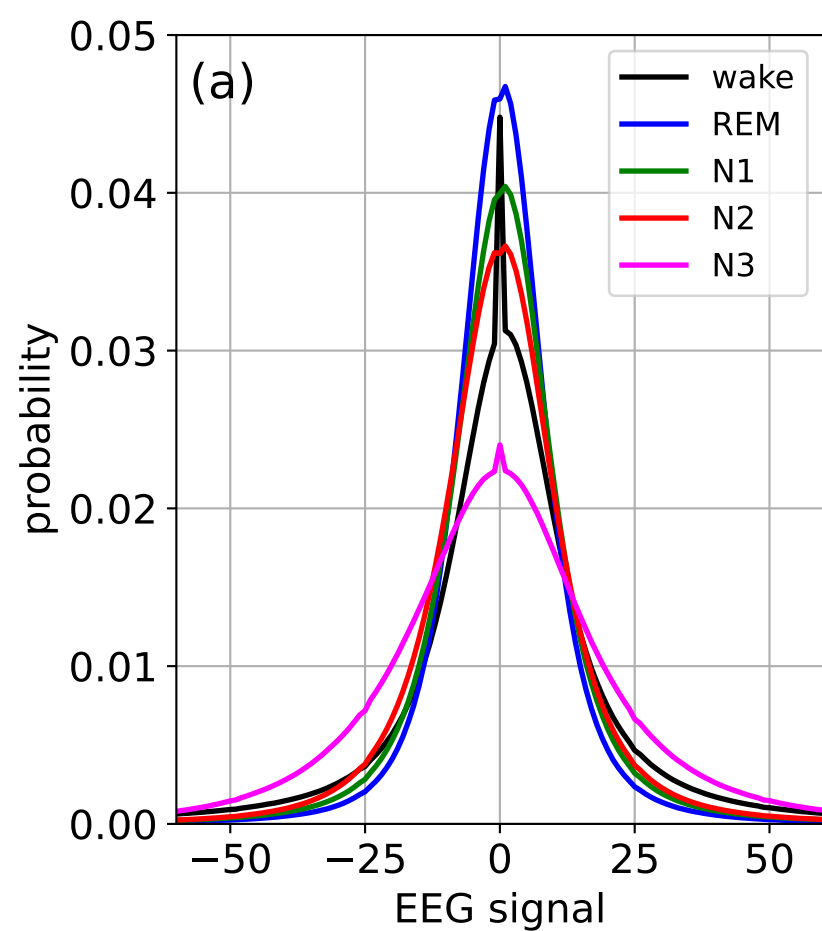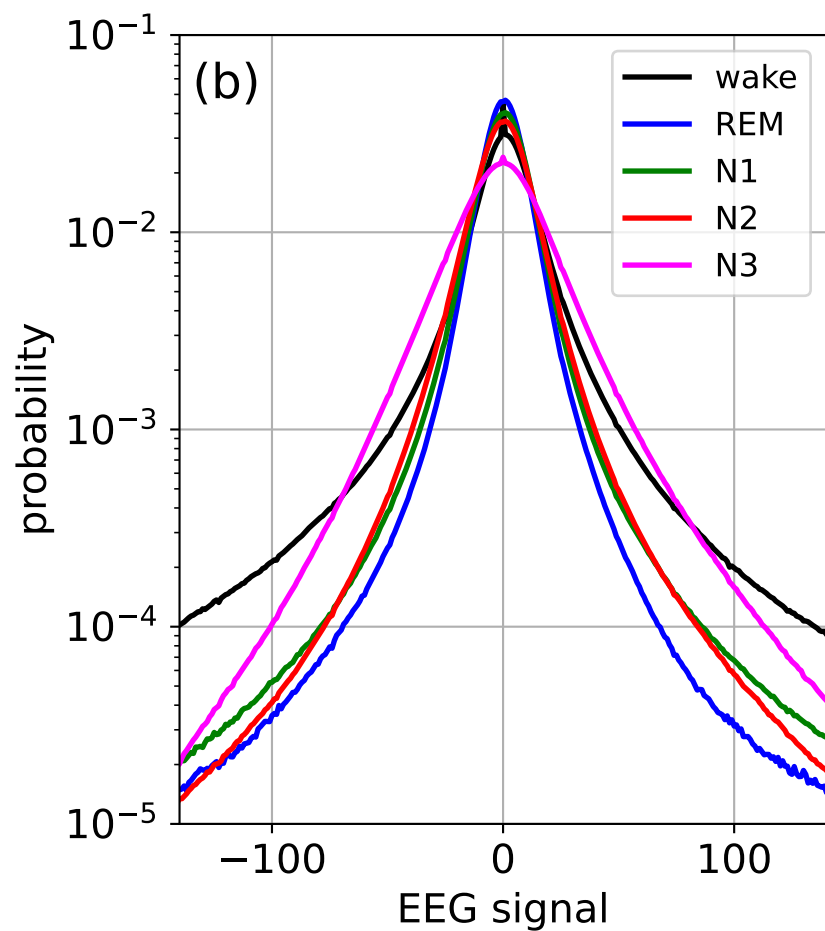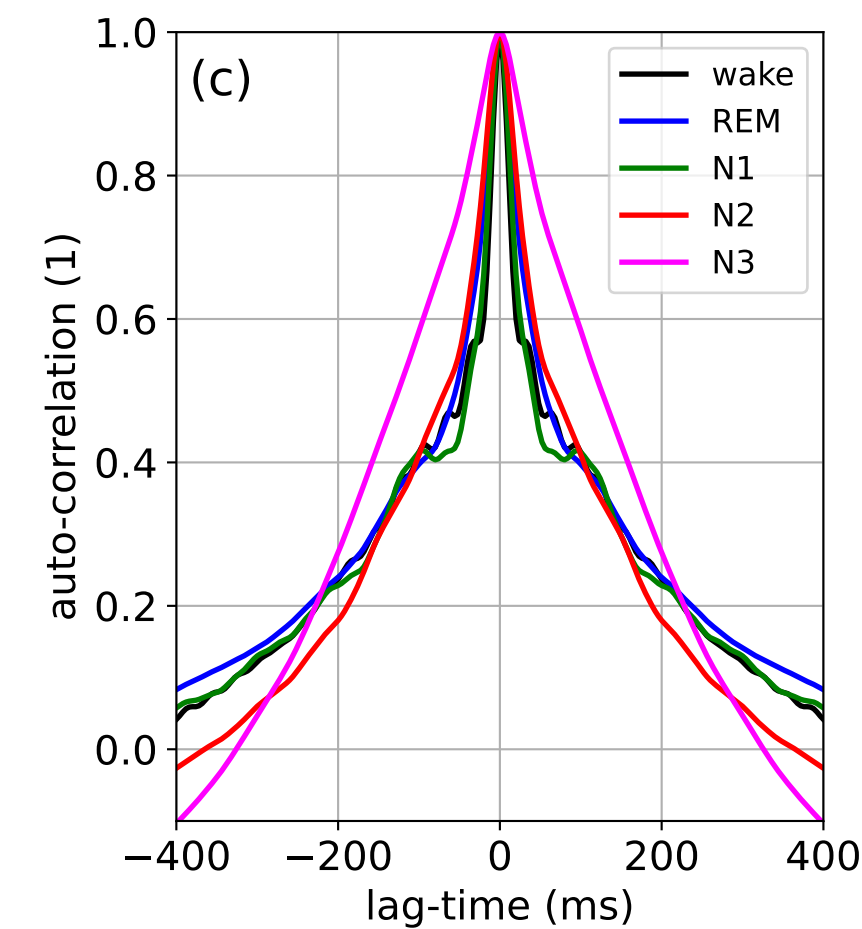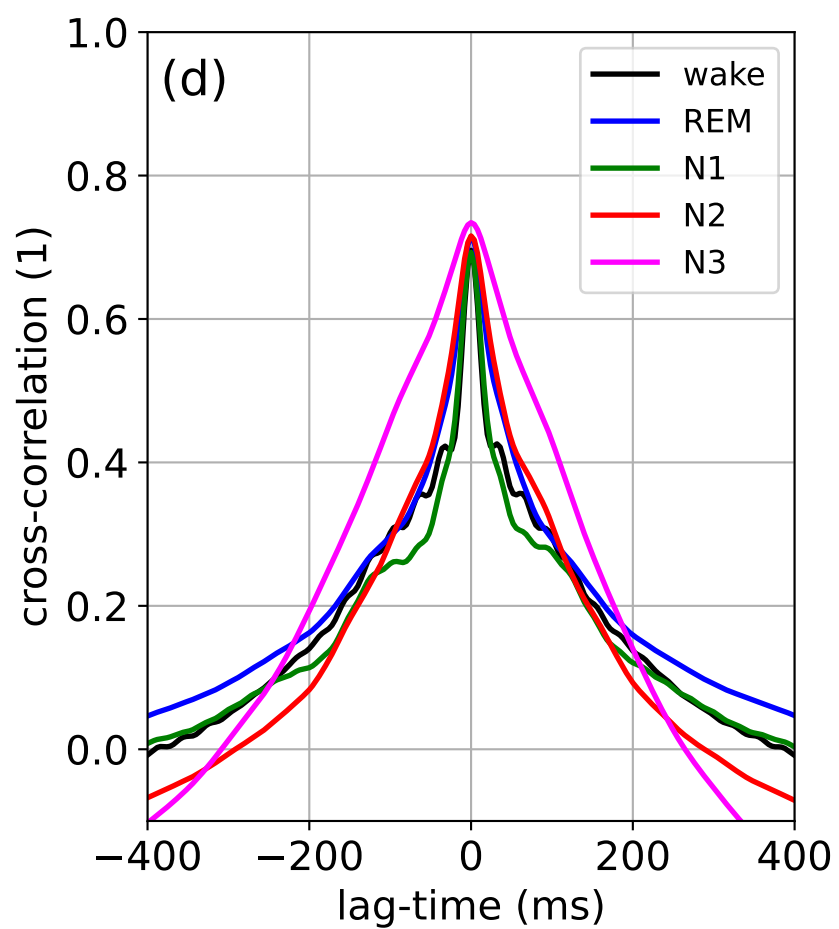

Supplement: Supplementary file 3 — Supplementary Data 1 [file 42003_2021_2912_MOESM3_ESM.zip › SupplementaryData1/Prg_Figure1/FIGURES/EEG.pdf]

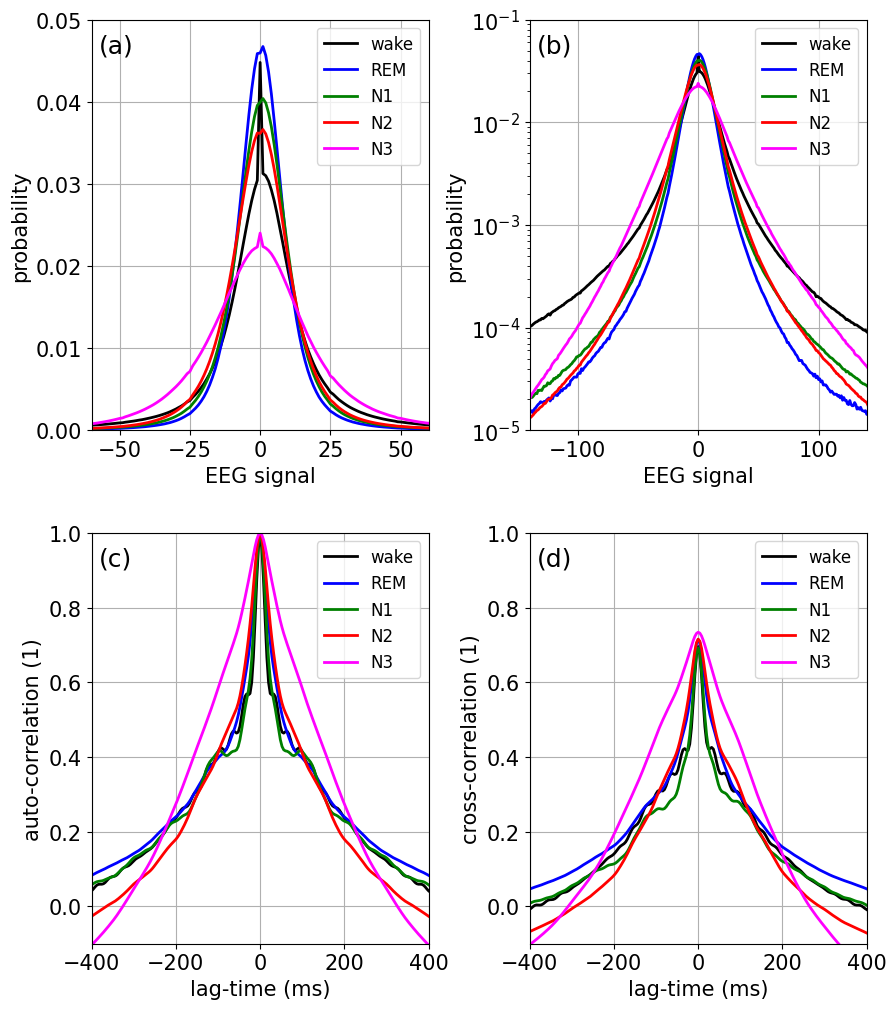

Supplement: Supplementary file 3 — Supplementary Data 1 [file 42003_2021_2912_MOESM3_ESM.zip › SupplementaryData1/Prg_Figure1/FIGURES/EEG.png]

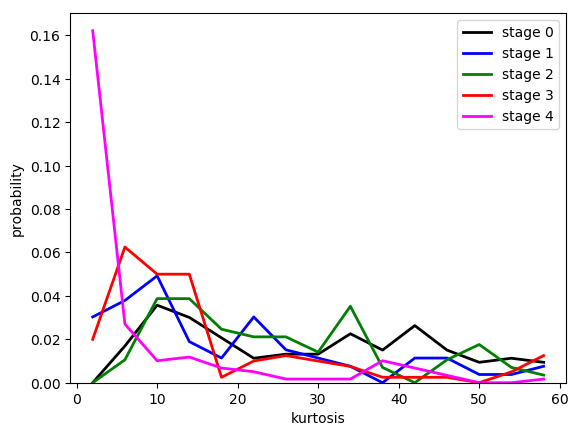

Supplement: Supplementary file 3 — Supplementary Data 1 [file 42003_2021_2912_MOESM3_ESM.zip › SupplementaryData1/Prg_Figure1/PDF/dist_kur.png]

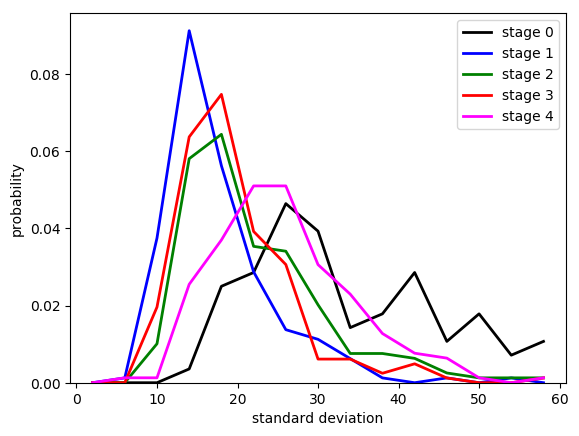

Supplement: Supplementary file 3 — Supplementary Data 1 [file 42003_2021_2912_MOESM3_ESM.zip › SupplementaryData1/Prg_Figure1/PDF/dist_std.png]

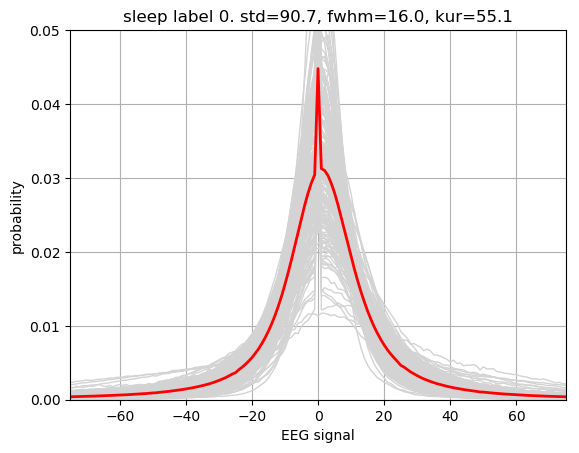

Supplement: Supplementary file 3 — Supplementary Data 1 [file 42003_2021_2912_MOESM3_ESM.zip › SupplementaryData1/Prg_Figure1/PDF/Stage_0.png]

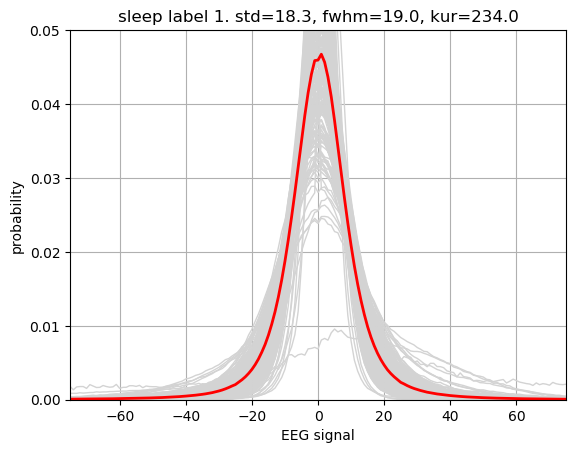

Supplement: Supplementary file 3 — Supplementary Data 1 [file 42003_2021_2912_MOESM3_ESM.zip › SupplementaryData1/Prg_Figure1/PDF/Stage_1.png]

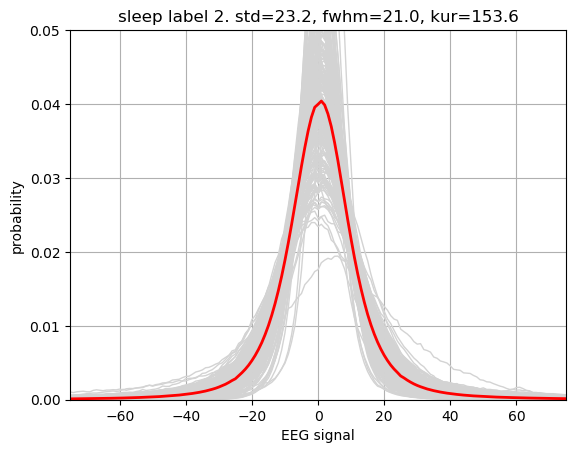

Supplement: Supplementary file 3 — Supplementary Data 1 [file 42003_2021_2912_MOESM3_ESM.zip › SupplementaryData1/Prg_Figure1/PDF/Stage_2.png]

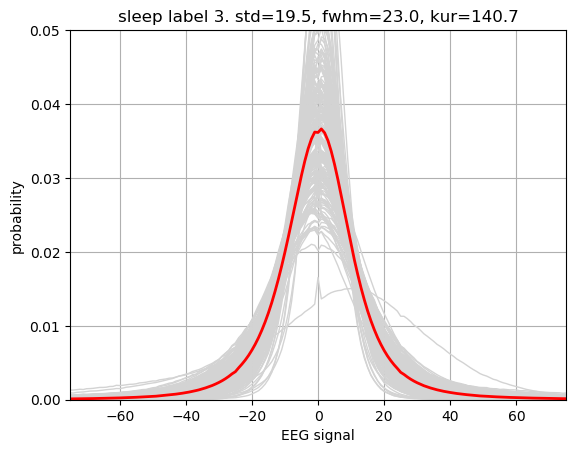

Supplement: Supplementary file 3 — Supplementary Data 1 [file 42003_2021_2912_MOESM3_ESM.zip › SupplementaryData1/Prg_Figure1/PDF/Stage_3.png]

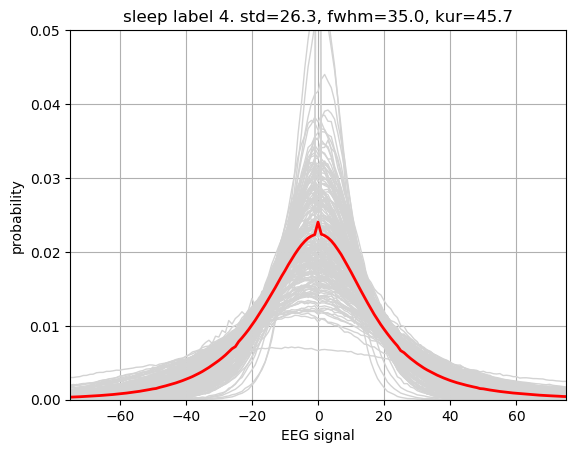

Supplement: Supplementary file 3 — Supplementary Data 1 [file 42003_2021_2912_MOESM3_ESM.zip › SupplementaryData1/Prg_Figure1/PDF/Stage_4.png]

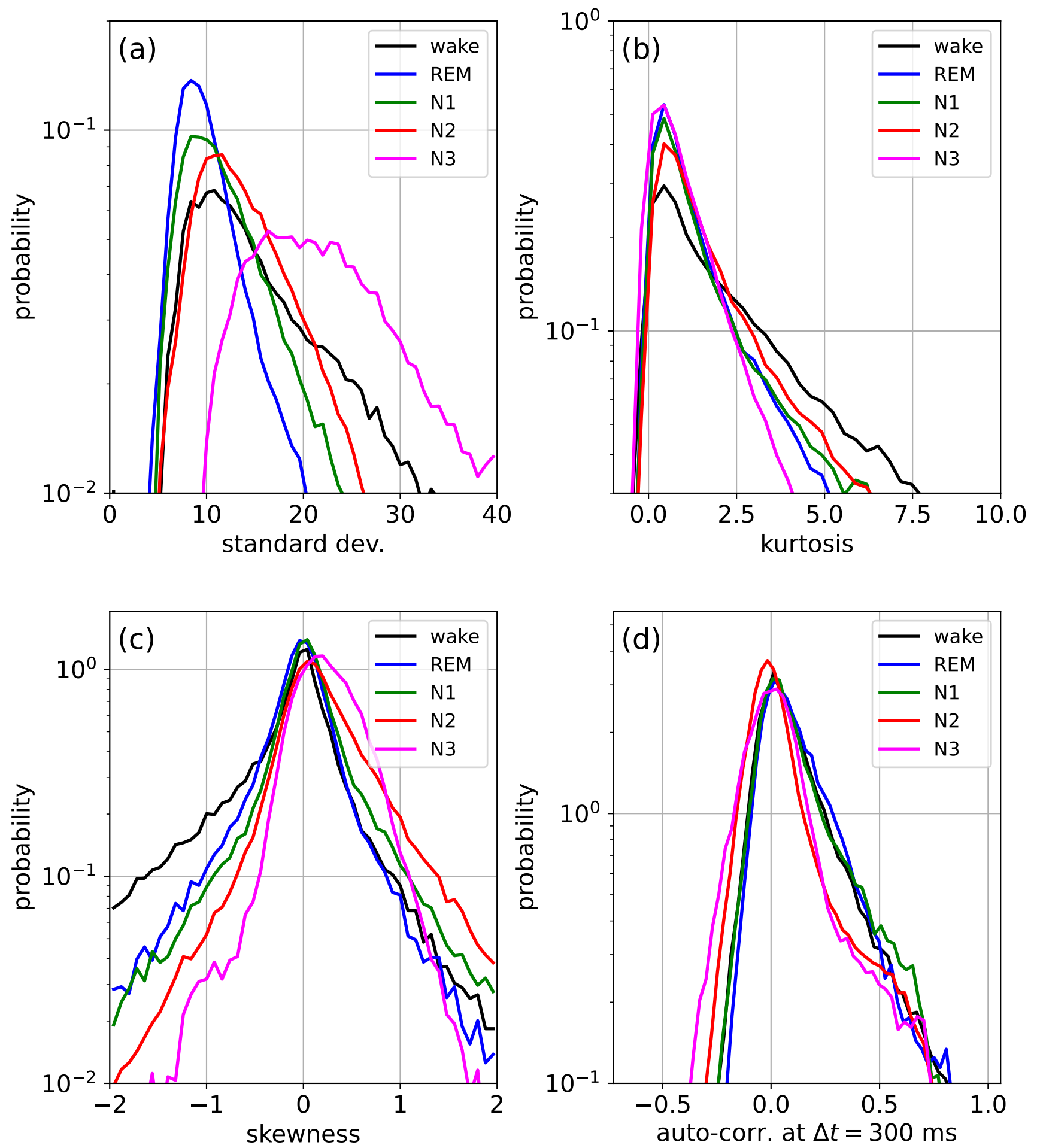

Supplement: Supplementary file 3 — Supplementary Data 1 [file 42003_2021_2912_MOESM3_ESM.zip › SupplementaryData1/Prg_Figure3/FIGURES/HypParDist.pdf]

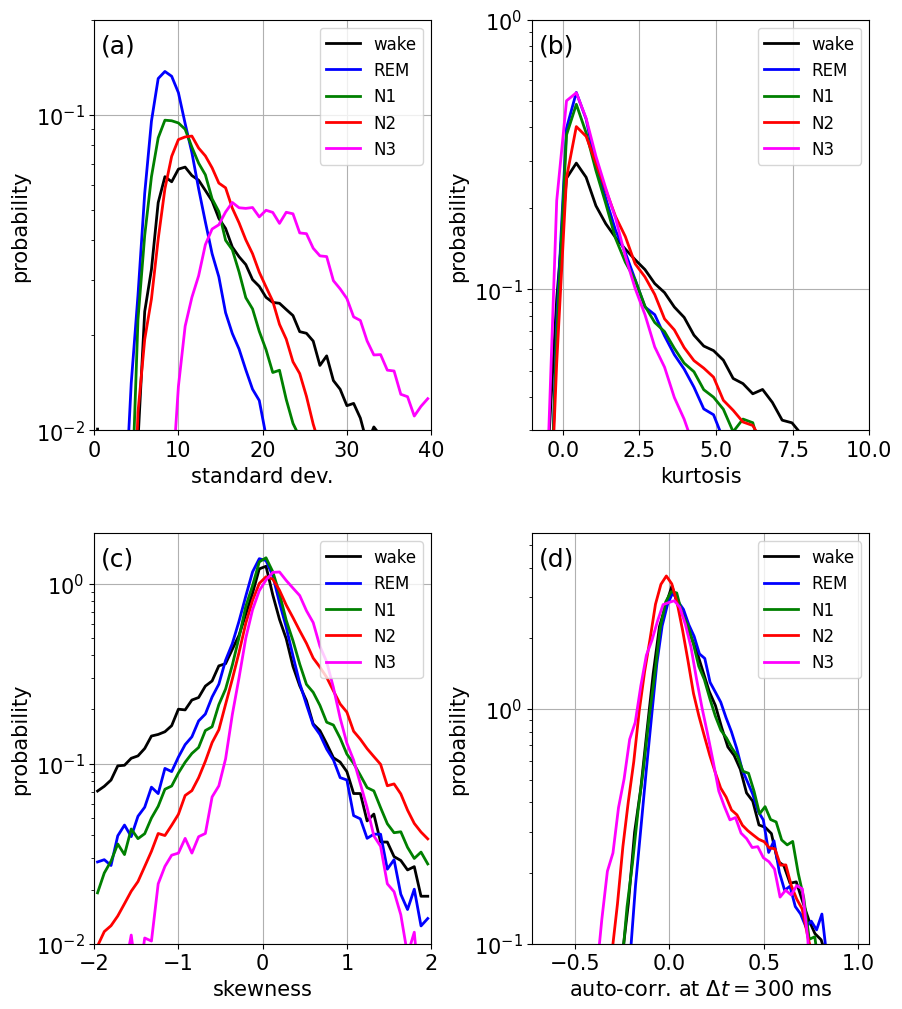

Supplement: Supplementary file 3 — Supplementary Data 1 [file 42003_2021_2912_MOESM3_ESM.zip › SupplementaryData1/Prg_Figure3/FIGURES/HypParDist.png]

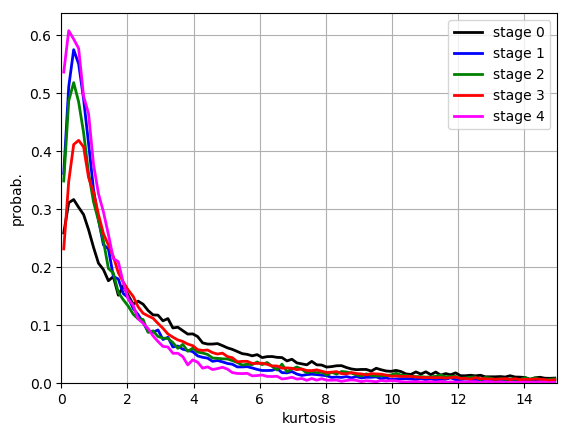

Supplement: Supplementary file 3 — Supplementary Data 1 [file 42003_2021_2912_MOESM3_ESM.zip › SupplementaryData1/Prg_Figure3/PAR/kurHist.png]

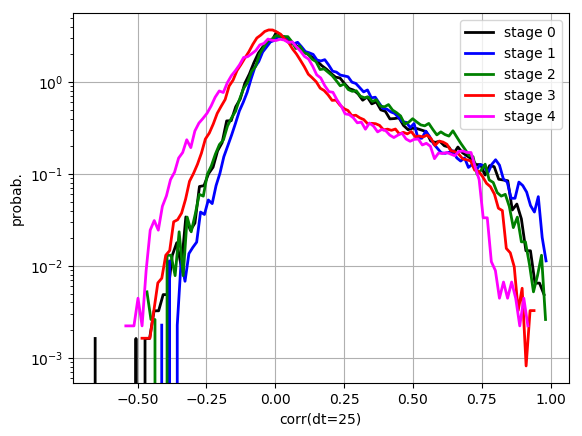

Supplement: Supplementary file 3 — Supplementary Data 1 [file 42003_2021_2912_MOESM3_ESM.zip › SupplementaryData1/Prg_Figure3/PAR/s25Hist.png]

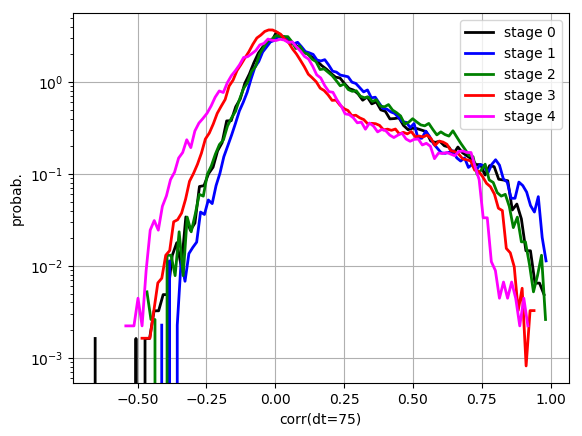

Supplement: Supplementary file 3 — Supplementary Data 1 [file 42003_2021_2912_MOESM3_ESM.zip › SupplementaryData1/Prg_Figure3/PAR/s75Hist.png]

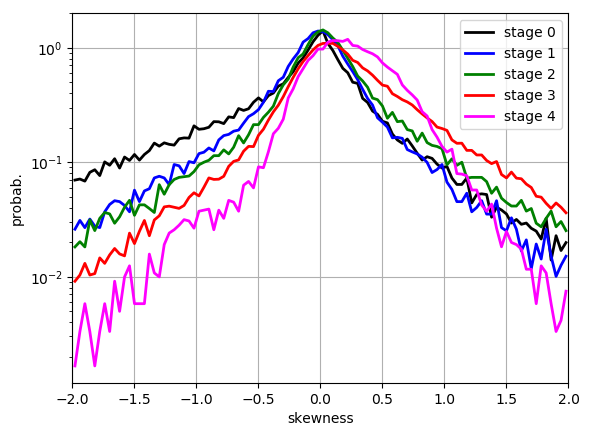

Supplement: Supplementary file 3 — Supplementary Data 1 [file 42003_2021_2912_MOESM3_ESM.zip › SupplementaryData1/Prg_Figure3/PAR/skeHist.png]

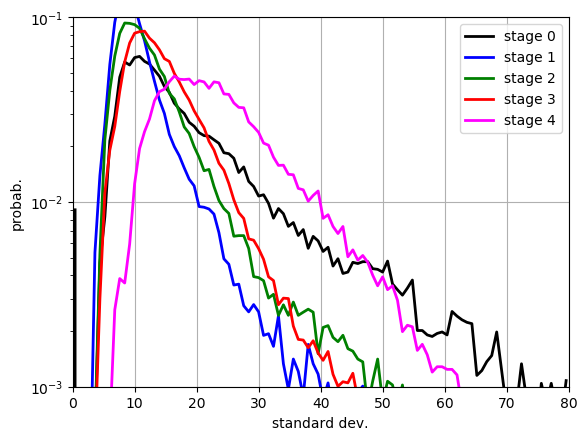

Supplement: Supplementary file 3 — Supplementary Data 1 [file 42003_2021_2912_MOESM3_ESM.zip › SupplementaryData1/Prg_Figure3/PAR/stdHist.png]

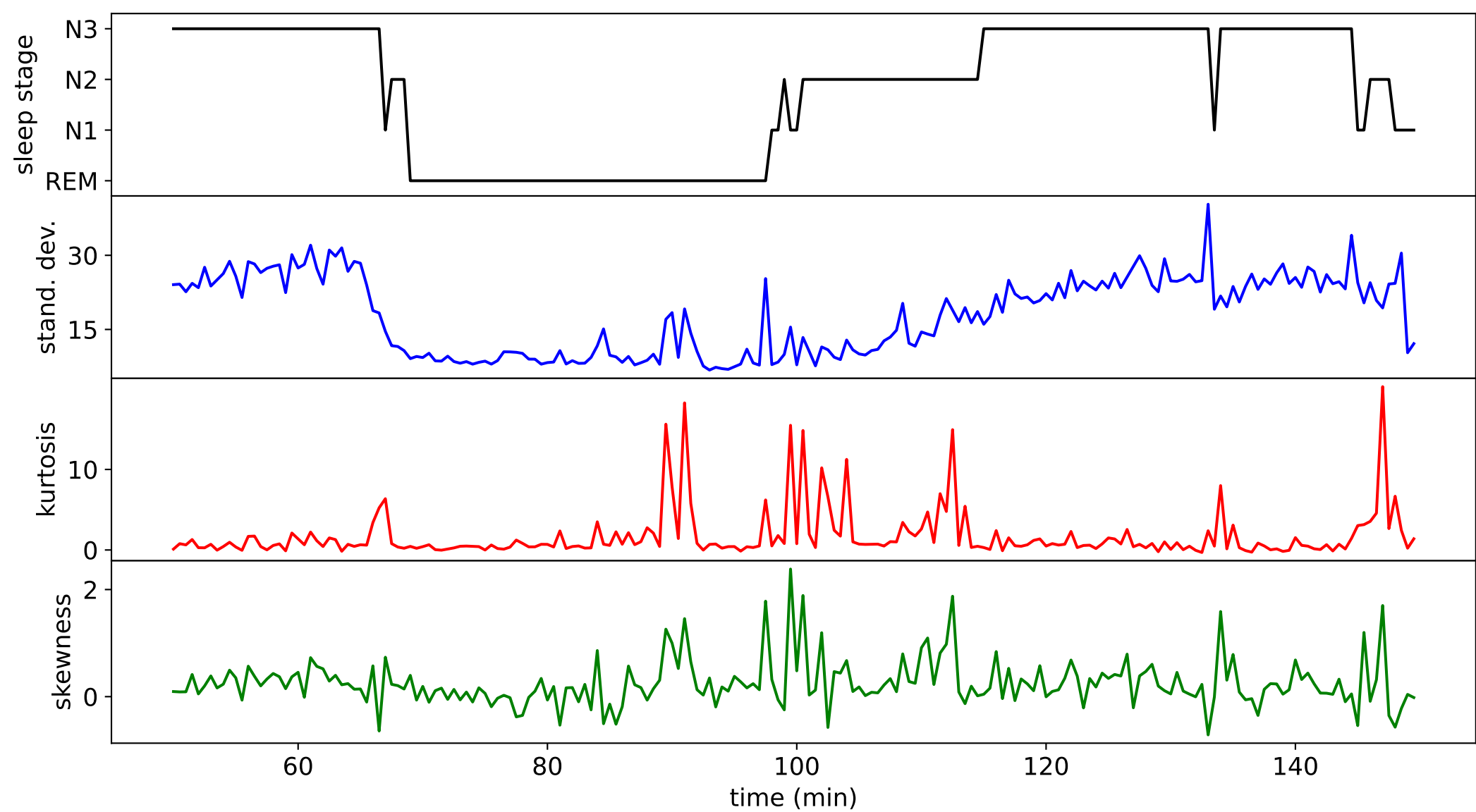

Supplement: Supplementary file 3 — Supplementary Data 1 [file 42003_2021_2912_MOESM3_ESM.zip › SupplementaryData1/Prg_Figure4/hypertrace.pdf]

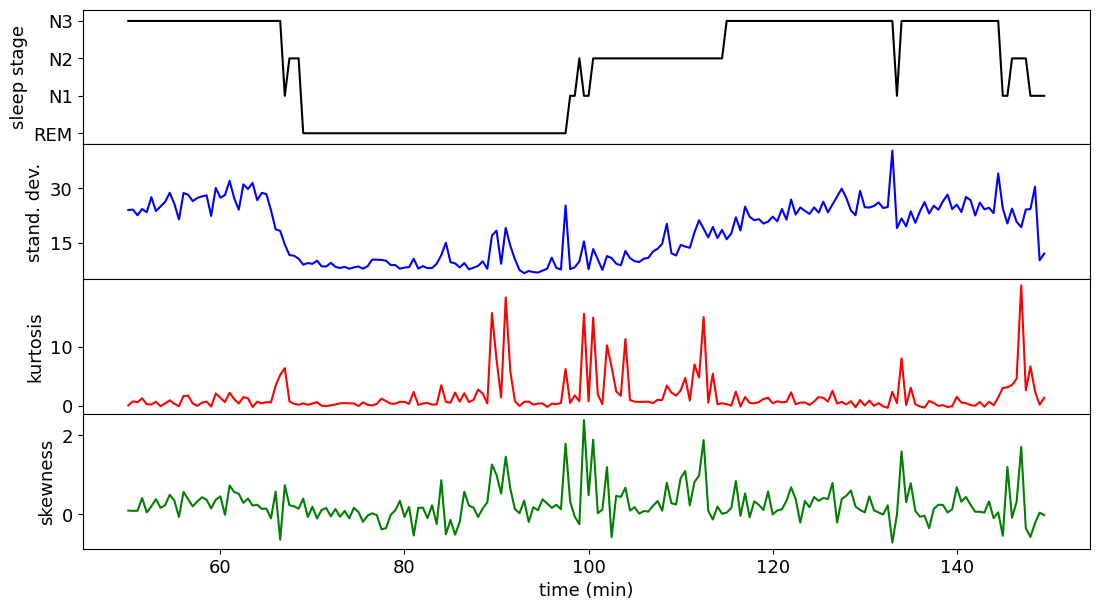

Supplement: Supplementary file 3 — Supplementary Data 1 [file 42003_2021_2912_MOESM3_ESM.zip › SupplementaryData1/Prg_Figure4/hypertrace.png]

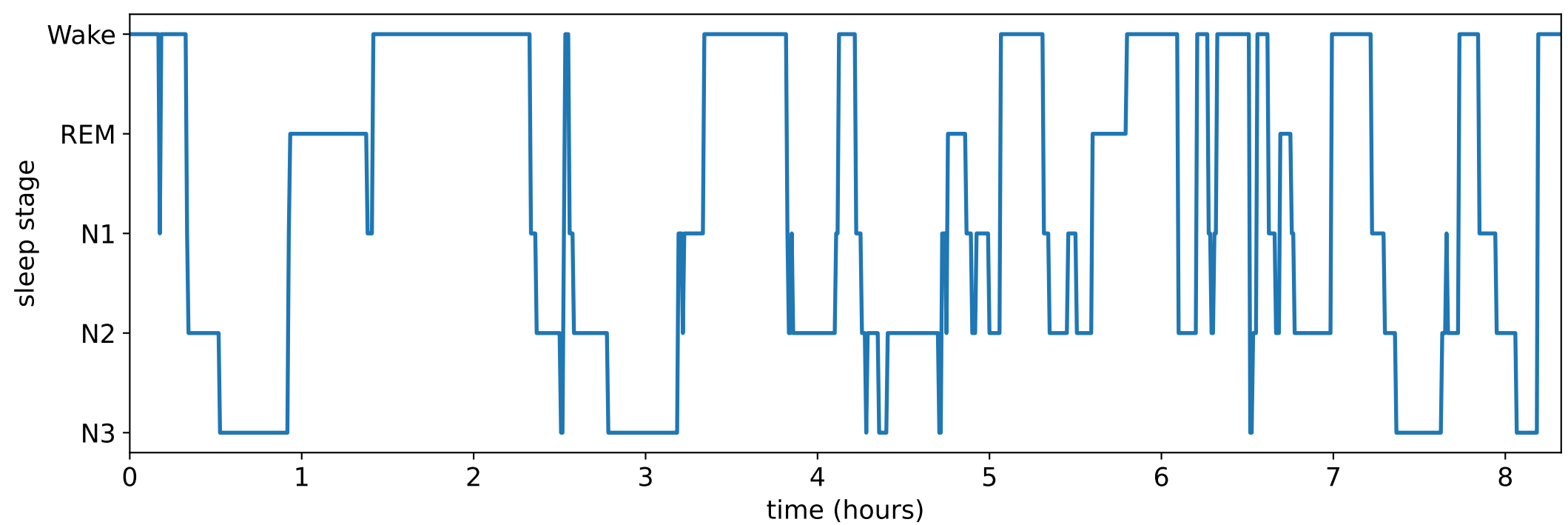

Supplement: Supplementary file 3 — Supplementary Data 1 [file 42003_2021_2912_MOESM3_ESM.zip › SupplementaryData1/Prg_Figure5/markov.pdf]

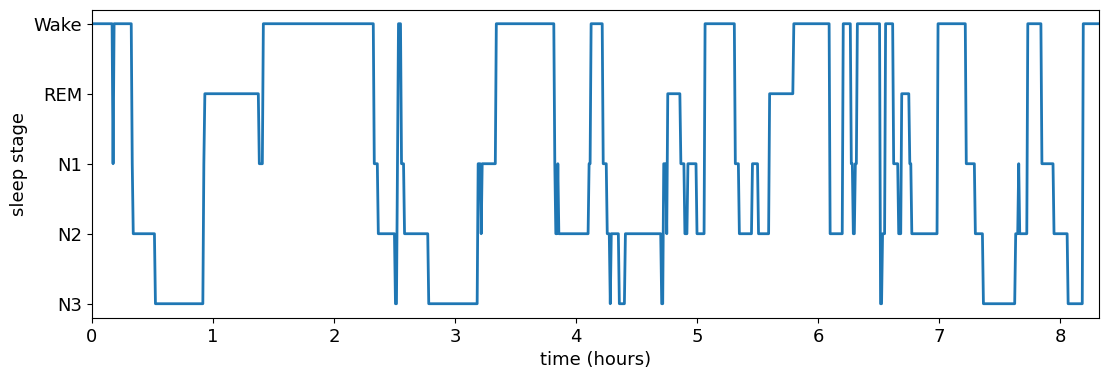

Supplement: Supplementary file 3 — Supplementary Data 1 [file 42003_2021_2912_MOESM3_ESM.zip › SupplementaryData1/Prg_Figure5/markov.png]

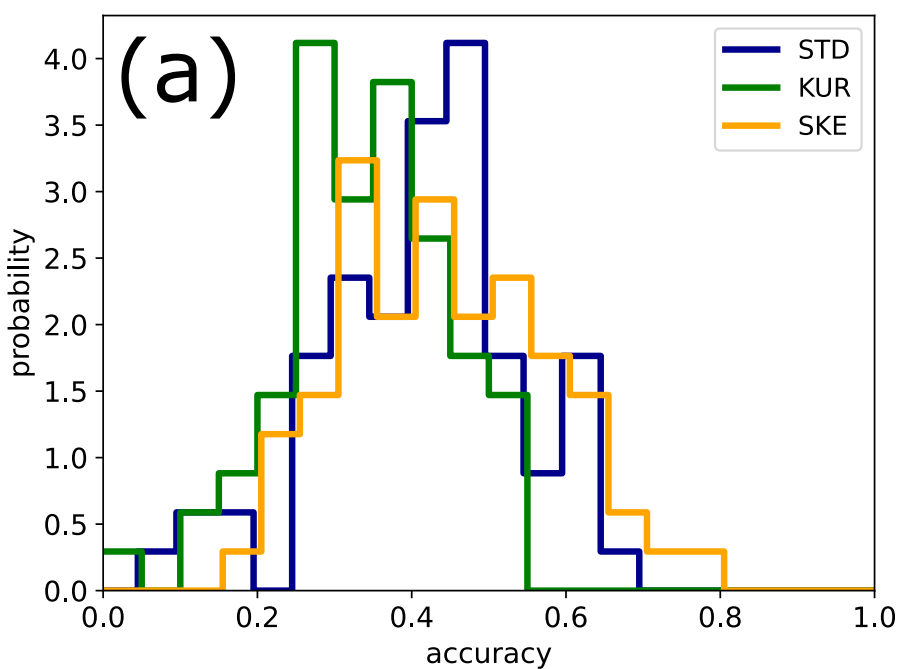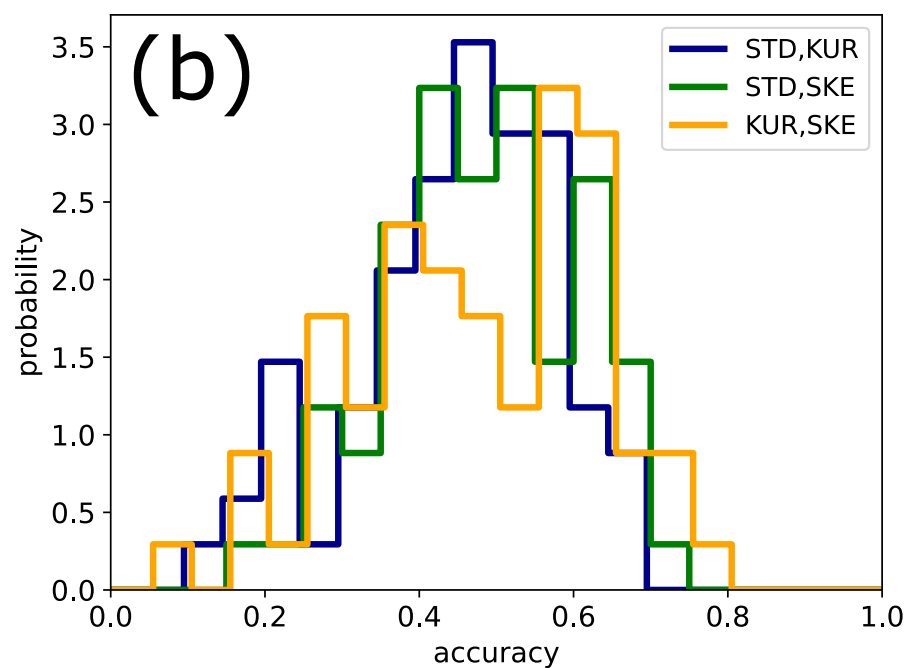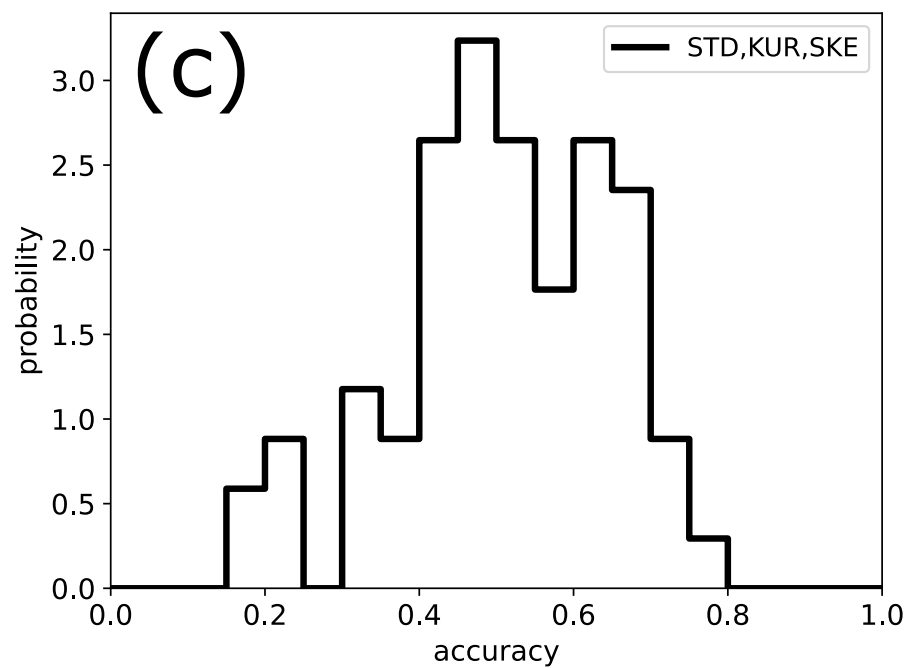

Supplement: Supplementary file 3 — Supplementary Data 1 [file 42003_2021_2912_MOESM3_ESM.zip › SupplementaryData1/Prg_Figure7/FIGURE_S2/FigS2.pdf]
